# A multi-contextual examination of non-school friendships and their impact on adolescent deviance and alcohol use

**DOI:** 10.1371/journal.pone.0245837

**Published:** 2021-02-10

**Authors:** Rupa Jose, John R. Hipp, Carter T. Butts, Cheng Wang, Cynthia M. Lakon

**Affiliations:** 1 Department of Psychological Science, University of California Irvine, Irvine, California, United States of America; 2 Department of Criminology, Law and Society, University of California Irvine, Irvine, California, United States of America; 3 Department of Sociology, University of California Irvine, Irvine, California, United States of America; 4 Department of Sociology, Wayne State University, Detroit, Michigan, United States of America; 5 Program in Public Health, University of California Irvine, Irvine, California, United States of America; University of New Mexico, UNITED STATES

## Abstract

Despite decades of research on adolescent friendships, little is known about adolescents who are more likely to form ties outside of school. We examine multiple social and ecological contexts including parents, the school, social networks, and the neighborhood to understand the origins and health significance of out of school ties using survey data from the National Longitudinal Study of Adolescent to Adult Health (*N* = 81,674). Findings indicate that out of school (more than in-school) friendships drive adolescent deviance and alcohol use, and youth with such friends tend to be involved in school activities and are central among their peer group. This suggests that intervention efforts aimed at reducing deviance and underage drinking may benefit from engaging youth with spanning social ties.

## Introduction

A large body of research has demonstrated that adolescents’ social ties are important for understanding which youth are likely to become involved in risky health behaviors. Adolescent friendships are forged across and within numerous social contexts, including the school, neighborhood, during extracurricular activities, or through close personal ties. Research on in-school friendships has historically dominated the literature, even though work by Kiesner [1, 2] and Ennett [3, 4] find that friendships and peers outside of school can be especially influential on adolescent delinquency and alcohol use. The role of adolescents’ social networks on their deviance and alcohol use behavior therefore requires understanding the nature of out of school friendships.

In a study of Swedish adolescents, Kiesner and colleagues [1] found that where a friendship was formed mattered, with adolescents who formed friendships at a youth center or in their neighborhood self-reporting the highest levels of delinquency. This finding is similar to earlier research by Dishion, Andrews, and Crosby examining American delinquent friendship dyads using information gathered from adolescent boys. Across 135 delinquent friendship dyads, it was found that 73% met at school or during a structured activity and 17% met either in their neighborhood or during an unstructured activity [5]. Adolescent alcohol use is also similarly believed to be affected by outside of school relationships. As reported by Ennett and colleagues [3], youth who indicated more out-of-network (different grade or school) friends at age 11 had significantly higher odds of alcohol use at 11 and 13 years. In a second study by Ennett et al. [4], neighborhood modeling (i.e., the average alcohol misuse of adolescents in the neighborhood) was found to positively predict adolescent alcohol misuse. These studies link out of school peers with adolescent deviance and alcohol use but provide limited insight on out of school friendships.

In the current study, we focus on out of school friendships defined broadly as friends not enrolled in the same school. Guided by the larger delinquency and substance use literature we also connect friendship nominations to adolescent deviance (rule-breaking behavior) and alcohol use behavior. Specifically, we aim to answer the question “What personal, network, and neighborhood characteristics are associated with adolescent out of school friendships?” and “Are out of school friendship ties predictive of adolescent deviance or alcohol use behavior, above and beyond in school friendships and other relevant covariates?” These questions are examined using a nationally representative sample of American adolescents from the National Longitudinal Study of Adolescent to Adult Health (Add Health) dataset.

### Friendship networks, delinquency, and alcohol use

A large body of research has explored the relationship between adolescents’ social networks and delinquent and alcohol using behavior. Studies have found a positive relationship between the number of delinquent friends or rate of alcohol use within an adolescent’s peer group and his/her level of delinquency [6, 7] or frequency of alcohol use [8]. A general theoretical question that arises from this literature is whether adolescents have friends that are similar to them in delinquency or alcohol use behavior because of a selection effect (i.e., delinquent or alcohol using youth form social ties with other delinquent or alcohol using youth) or because of an influence effect (i.e., youth become more delinquent or drink more over time because they are influenced by the delinquent or alcohol using behavior of their friends).

Findings from SIENA (Simulation Investigation for Empirical Network Analysis; [9]) studies which focus on the in-school network indicate that adolescents select friends that are similar to themselves in terms of gender, race/ethnicity, socioeconomic status, delinquency engagement [10, 11] and alcohol use [12–14]. Adolescents also tend to change their behavior over time to better match the delinquent or alcohol using behavior [12–14] of their peer group, with influence effects moderated by gender similarity and the level of tie reciprocity [11, 15]. Bounding the network to the school however ignores the potentially influential friendships that exist outside of school. And though schools are an important context for tie formation, they are not the sole context. Of emerging importance are studies indicating that ties which form outside of the school are associated with adolescent delinquent behavior [16, 17] and substance use [18, 19]. It is therefore expected that adolescents who have more out of school friendships will report greater deviance and alcohol use.

Two studies that directly assessed non-school peers have been conducted by Jose et al. [16] and Tucker et al. [19]. In terms of the Jose et al. [16] study, friendship ties and delinquency engagement were examined with respect to the number of out of school friendships and neighborhood attributes. Results indicated that adolescents in small schools who reported more out of school friendships also reported more delinquent behaviors over time, while adolescents in large schools were more likely to befriend other adolescents from equally disadvantaged neighborhoods [16]. Furthermore, adolescents in both kinds of schools were more likely to form friendships with peers who lived closer to their home of residence [16]. This maps onto early work by Weerman [17] on street youth groups which found that adolescents who joined or left an informal (unsupervised) youth group consisting of peers aged 12–25 who socialized in public spaces (the “streets”) were significantly more likely to increase their delinquent behavior over time. Though the youth group was not explicitly operationalized as out of school ties, the wider age range, the requirement that members spend “a lot” of time in public places outside a school setting, and the decision to measure the school influence separately, makes it plausible that these street youth groups proxy for non-school friendships. In the second study by Tucker et al. [19], having only out of school friends was associated with subsequent marijuana use initiation but not binge drinking initiation for adolescents 12–19 years. While these studies approximate or explicitly measure out of school friendships, they do not consider the myriad of interpersonal and contextual factors that may be important for *bringing about* out of school ties, as well as adolescent deviance or alcohol use.

### Where do out of school ties come from, and what are their consequences for deviance and alcohol use?

Nascent research that has focused on out of school ties has typically asked how they relate to deviance or alcohol use, not fully exploring the characteristics and environments underlying out of school friendships. Due to the apparent significance of this type of tie, it is useful to explore where such ties come from, and why some adolescents have more of these ties than others. Settings that promote informal and unstructured socializing have been previously theorized or shown to provide increased opportunities for out of school friendships and deviancy [20, 21] though other settings also likely matter. Four main social dimensions are considered given their hypothesized importance: 1) parents’ level of monitoring and emotional support; 2) the structural context of the school, along with the school organizations in which the adolescent participates; 3) the school network and the adolescent’s position within it; and 4) the neighborhood. Each of these are discussed in turn.

### Parental support and parental monitoring

One of the first influential agents in a child’s life is their parents. During adolescence, the quality of the parent-child relationship carries a powerful impact on development [22, 23], delinquency engagement (for review see [24]), and alcohol use [25]. Two facets of the parent-child relationship that bear importantly on adolescent friendships and behavior are parental support and parental monitoring.

Though no known studies have examined parental support or monitoring in relation to the number of out of school friendships, several studies have found robust relationships between parental factors and in-school friendships. Evidence from prior studies suggest that exposure to high levels of parental support can result in an increased number of in-school friends [13, 26] whereas high levels of parental monitoring can decrease the number of substance using in-school friendships (i.e., friends that smoke) reported over time. Given the relationship between out of school friendships and delinquency [16, 19, 20], and the fact that out of school friendships develop in a variety of different settings with varying degrees of structure and supervision [1, 5], we posit that adolescents will endorse more out of school friendships when in the presence of low support, low monitoring parents.

Adolescents with a supportive parent they can trust and seek understanding from are less pressured by peers, report higher self-esteem, and engage in fewer deviant behaviors (i.e., alcohol use and delinquency; [27]). In addition, adolescents with parents high in warmth, love, and communication abilities engage in less violent or property delinquency; an association that remains strongly significant after accounting for parental monitoring and parental involvement [28]. Low parental warmth has also been associated with increased binge drinking (youth aged 11–21 years; [29]). When just considering maternal support, high levels of maternal support have a negative, direct effect on delinquency engagement as well as an indirect effect on delinquency engagement by decreasing deviant peer friendships [30]. Likewise, maternal and paternal support were both found to buffer the effect of affiliation with drug-using peers on adolescent girls’ alcohol use [31]. It is therefore anticipated that access to supportive parents will have a negative effect on adolescent deviance and alcohol use.

The relationship between parental monitoring and deviance is somewhat more complex. Research finds that adolescents whose parents monitor their whereabouts and their peer associates report a lower initial rate of delinquency and fewer delinquent behaviors over time compared to adolescents with low monitoring parents [32]. However, the efficacy of parental monitoring seems to vary by context. As reported by Osgood and Anderson [33], adolescents in a school with students whose parents are high monitors engage in less unstructured socializing, which is associated with less delinquent behavior over a 12-month period even if their own parents are not involved in monitoring their whereabouts. Tilton-Weaver and colleagues [34] also found that parent monitoring rules were effective at reducing the selection of delinquent peers among older adolescents (15–18 years) and early adolescents (9–11 years) who did not feel ‘overcontrolled,’ but not middle adolescents (12–14 years). A review of studies on adolescent alcohol use found that increased parental monitoring was associated with early alcohol use initiation but less alcohol use over time [25].It is therefore anticipated that adolescents with high monitoring parents will report low levels of deviance and alcohol use.

### School structure and organizations

Schools are places where adolescents can befriend peers and engage in either prosocial or antisocial activities. For this reason, we consider the theoretical merit of school-based factors such as extracurricular activities, school type, and years enrolled in school, in relation to out of school friendships. We also consider the importance of years enrolled in school and school attachment for adolescent deviance/alcohol use.

School clubs and activities can give adolescents access to prosocial peers, provide adult supervision, help in social and communication skills development, provide exposure to behavioral norms, and encourage school attachment [35]. We therefore expect that greater participation or integration in school clubs or activities will be associated with an expansive school-centric peer network, resulting in less out of school ties.

The type of school may impact friendship formation outside of school due to geographic or population differences. For example, public schools have a more racially/ethnically diverse student body, more students with limited English-speaking abilities, a larger number of students, and bigger class sizes on average than private schools [36]. Youth from diverse school or neighborhood settings moreover tend to select “same-ethnic” over “cross-ethnic” friendships [37]. For private school students, friends forged in the neighborhood or through community activities may represent more of their friend group if they increase opportunities for “same-ethnic” friendships or provide easy access to peers from similar social backgrounds. Being that private schools are smaller in size, less diverse, and often have students coming from demographically different (and potentially distal) neighborhoods, out of school ties are expected to be greater for private school students compared to public school students.

The number of years a student is enrolled in school can also affect both the number of out of school friendships adolescents have and their level of deviance/alcohol use. Students who are new to a school may report more out of school friendships than in-school friendships because in-school friendships, especially the intimate and loyal friendships desired during adolescence, often need time to develop [38]. Therefore, we predict that the more years an adolescent has been in a school, the less likely they are to report out of school friendships. Students who have been in a school longer may also report higher levels of school connectedness and school engagement, a factor positively associated with future well-being [39] and negatively associated with serious delinquency and problem substance use (e.g., drug and/or alcohol use; [40–42]). As such, we predict that adolescents with a greater attachment to their school, along with those who have spent more years at a school, will report less deviance and alcohol use.

### School networks

An adolescent’s position within the school network can impact their ability to maintain ties outside of the school context. It is expected that adolescents who are particularly central in their school network (based on network measures) will have fewer ties outside of the school due to ease of fostering in-school friendships. Being a friend and certainly ‘best friend’ can be a time-consuming process, often requiring a commitment to engage in shared activities, acts of reciprocity, or merely “be there” for another person [43]. Friendships are therefore more likely to occur and be maintained when adolescents belong to the same school as opposed to different schools [44].

Adolescents who are members of close in-school groups that have a high density of ties among group members are expected to have fewer friendship ties outside the school due to the stress of maintaining multiple friendships [45]. For school networks that exhibit a relatively high density of ties among adolescents, a structural effect is expected in which adolescents who are members of such schools have fewer ties outside of the school. Furthermore, schools where most ties are reciprocated or friendships are mutually endorsed suggest a close-knit school environment. Adolescents enrolled in close-knit schools may report few out of school friendships, if any.

### Neighborhoods

In school or in their neighborhood, adolescents tend to pick friends similar to themselves (homophily effect) [46]. As reported by Currarini, Jackson, and Pin [47] Asian, Black, and Hispanic students show significant bias in interacting with same-race peers, place greater value over same-race friendships, and are more likely to self-segregate in large educational settings. Moreover, as found by Mouw and Entwisle [48], friends are more likely than non-friends to be of the same socioeconomic background–with school friend dyads reporting similarity in parent education level and income. This “birds of a feather” explanation suggests that living in less homogenous neighborhoods, due to either high ethnic heterogeneity or income inequality, could result in adolescents endorsing fewer out of school friendships when compared to adolescents residing in single class neighborhoods where neighborhood and personal identity are aligned [49]. On the other hand, neighborhood residential stability (living in a neighborhood that is more stable in terms of resident influx) can provide adolescents with the opportunity to get to know and form relationships, over time, with their adult and juvenile neighbors [50]. Taken together, adolescents from residentially stable neighborhoods are expected have more out of school friends while adolescents from neighborhoods high in income inequality and ethnic heterogeneity are expected to have fewer out of school friends on average.

Spatial propinquity also has important consequences for tie formation among adolescents [16, 48]. Adolescents are more likely to form social ties with fellow schoolmates to whom they live closer. We therefore expect to observe a macro pattern such that when students in schools live, on average, closer to one another they are more likely to form in-school ties. Similarly, if students live in close spatial clusters to other students in the school, we expect that students will have more in-school ties and fewer out of school ties. For schools in which students live, on average, longer distances from the school, more opportunities for out of school ties are possible and therefore are anticipated.

Finally, deviant behavior tends to flourish in neighborhoods that are economically disadvantaged. One example of this comes from a study on boys in the Pittsburgh area which found that structural neighborhood differences corresponded to prevalence differences in the engagement of serious criminal offenses (attacking to seriously hurt or injure, selling drugs, etc.). For boys high in protective factors, or with a mix of protective and risk factors, living in socioeconomically disadvantaged neighborhoods was associated with higher offending rates in late adolescence (after age 12) compared to living in advantageous neighborhoods [51]. Findings from a review by Leventhal and Brooks-Gunn [52] also indicate that adolescents in low socioeconomic status or low income neighborhoods tend to report more delinquent behaviors and alcohol use than youth in other neighborhoods. Neighborhood disadvantage is therefore expected to be positively correlated with adolescent deviance and alcohol use.

### Current study

In this study, we concurrently explore out of school friendships and their relationship with adolescent health risk behaviors using the large, nationally representative dataset of Add Health adolescents. With few descriptive studies on adolescents’ non-school friendships, the current study helps explain the presence of such ties by examining the factors that predict out of school friendships. A multi-contextual approach is applied to determine which parental, peer, school, and or neighborhood attributes are significantly associated with an adolescent’s number of out of school ties. We also examined the associations between out of school ties and adolescent deviance/alcohol use while controlling for traditional predictors and in-school ties.

## Methods

### Sample

This study was reviewed and granted approval under exempt review by the Institutional Review Board at the University of California, Irvine. Add Health participants provided written informed consent for participation in all aspects of Add Health in accordance with the University of North Carolina School of Public Health Institutional Review Board guidelines that are based on the Code of Federal Regulations on the Protection of Human Subjects 45CFR46: http://www.hhs.gov/ohrp/humansubjects/guidance/45cfr46.html. Written informed consent was given by participants (or next of kin/caregiver) for their answers to be used in this study. The student data used in the present study comes from the first wave of Add Health known as the in-school sample. The in-school sample includes information on 90,118 adolescents enrolled in 6^th^ to 12^th^ grade from 1994 to 1995. To be eligible for the larger Add Health study, schools had to include at least 30 students and an 11^th^ grade. The final sample included 81,674 adolescents from 126 schools (age: *M* = 15.0, *SD* = 1.7). Sample descriptive statistics are presented in Table 1.

**Table 1:** Summary statistics of variables used in analyses (*N* = 81,674).

	Mean	Std. Dev	Min	Max
Deviance	0.002	0.323	-1.213	1.431
Alcohol usage	1.218	1.529	0	6
Ties outside school	1.330	2.084	0	10
Ties inside school	4.247	3.050	0	10
***Parental measures***				
Parental monitoring	0.003	0.116	-0.457	0.495
Parental support	0.008	0.276	-1.435	1.091
Education (mother)	3.655	1.271	0	6
***School clubs measures***				
Number of academic clubs	0.302	0.728	0	8
Number of sports clubs	1.067	1.474	0	13
Number of arts clubs	0.294	0.581	0	4
Number of other clubs	0.437	0.822	0	8
***School level variables***				
School dropout rate	12.631	15.284	0	100
Public school	0.954	0.209	0	1
Catholic school	0.036	0.187	0	1
Private school	0.009	0.097	0	1
Average distance to school	326,000	165,751	67921.450	1002970
Standard deviation of distance between students in school	466,840	224,756	151924.7	1453905
Average distance between students in school	471,414	231,006	103164.3	1359690
***School network measures***				
Density	0.417	0.097	0.195	0.832
Mutuality index	0.379	0.046	0.231	0.529
Size of school	1,131	716	30	3546
***Personal network measures***				
In-degree	4.547	4.826	0	36
Bonacich centrality	0.794	0.644	0.000	4.964
Personal network density	0.298	0.152	0.000	1
***Block group level variables***				
Economic inequality	-49.7	8049.3	-37105.38	41699.92
Concentrated disadvantage	-0.001	0.118	-0.462	0.753
Population density	2.762	3.273	0	87.516
Proportion Black	0.692	0.462	0	1
Proportion Latinx	0.732	0.443	0	1
Proportion Asian	0.641	0.480	0	1
Proportion Other	0.684	0.465	0	1
Racial/ethnic heterogeneity	0.866	0.341	0	1
Proportion foreign born	0.635	0.481	0	1
Residential stability	-0.024	0.976	-4.386	4.468
***Individual level variables***				
Female	0.495	0.500	0	1
Grade	9.582	1.616	6	12
White	0.535	0.499	0	1
Black	0.157	0.364	0	1
Latinx	0.039	0.193	0	1
Asian	0.044	0.205	0	1
Native American/Other/Mixed Race	0.208	0.406	0	1
Native born	0.904	0.295	0	1
School Attachment	10.412	3.064	1	15
Years in this school	2.440	1.378	1	6

### Dependent variables

#### Number of out of school friends

Adolescents were asked to nominate 5 male friends (listed simply as “First Male Friend”, “Second Male Friend”, etc.) and 5 female friends using IDs found on a roster list provided by project staff. The roster list included the names of students in the adolescent’s school (sample school) and adolescent’s sister school. If a friend’s name was not on the roster list, adolescents could select from the following options: “nominated friend doesn’t go to sister or sample school,” “nominated friend goes to sister school—not on list,” and “nominated friend goes to sample school—not on list.” *Out of school friends* captures the number of people the adolescent nominated as a friend from outside the school, ranging from 0 to 10 friends. Friendship nominations that were deemed out of school friendships included friends enrolled in the adolescent’s sister school but not their own and friends enrolled in neither school. The number of out of school friends (“ties outside school”) was directly related to the number of in-school ties (“ties inside school”). For example, a student who nominated 10 friends, with 3 friends enrolled at the local sister school and the rest in their own school, would have 3 ties outside the school and 7 ties inside the school.

#### Adolescent deviance

Adolescents were asked to self-report on how often they lied to their parents or guardians, skipped school without an excuse, or got into a physical fight in the last 12 months using either a 5-point (0 = never to 4 = more than 7 times) or 7-point (0 = never to 6 = nearly everyday) rating scale. Confirmatory factor analysis was used to generate a factor score variable which represented an adolescent’s level of deviance over the past year. The items used to construct our measure of adolescent deviance have also been used by others examining deviance and delinquency; see [16] and [53].

#### Adolescent alcohol use

Adolescents were asked to self-report on how often they drank beer, wine, or liquor in the last 12 months using a 7-point rating scale (0 = never to 6 = nearly everyday).

### Independent variables

We captured the parental context with two measures. *Parental monitoring* was based on several questions regarding the extent that parents made decisions for adolescents in having a weekend curfew, weekday bedtime, the amount of time they could watch TV, what they could watch on TV, and who they could hang out with. Adolescents were also asked to report on whether their parents were there when they left for school, came home, during bedtime, or while eating dinner. Responses were coded such that adolescents with parents who made decisions for them and had their parents present for various activities were considered to have high monitoring parents. Confirmatory factor analysis was used to generate a factor score variable representing adolescent perceptions of parental monitoring. *Parental support* was measured as the average parental support given to the adolescent by their mom and dad, based on adolescent reports of their biological and/or residential parents. Specifically, adolescents rated the degree they found either parent as warm and loving, caring, felt close to them, communicated well with them, and had a good relationship with them. A single confirmatory factor analysis was also used to generate two factor score variables representing adolescent perceptions of their mother’s and father’s emotional support. The mean of mother and father support (indicated by factor scores) created our measure of parental support. We assessed the model fit of these latent variables (deviance, parental support, and parental monitoring) by estimating a confirmatory factor analysis model with all three latent variables simultaneously, and although the model fit was not perfect given the significant chi square value, the approximate model fit statistics were excellent (RMSEA = 0.017; CFI = 0.967).

In addition, *parent education (mother)* was used as a proxy for socioeconomic status and coded such that higher values indicated a higher level of educational attainment (0 = never went to school, 1 = 8^th^ grade or less, 2 = beyond 8^th^ but did not graduate from high school or went to business, trade, or vocational school instead of high school, 3 = high school graduate or completed a GED, 4 = went to business, trade, or vocational school after high school or went to college but did not graduate, 5 = graduated from college or university, and 6 = professional training beyond a four-year college). Parental education was collected at multiple assessment points on both parents (residential father and residential mother). However, the parent survey was often only filled out by the residential mother given Add Health procedure. Only in instances where a mother, stepmother, or female guardian was not present, could the survey be completed by a male respondent. For accuracy, education data from the parent survey (parent self-report) was used when available, with any missing values replaced by adolescent reports of residential mother’s educational attainment.

We constructed measures of the school context at the individual level. *Number of years in school* measures the number of years an adolescent has been enrolled in their given school using a 6-point scale where 1 = one year, 2 = two years, 3 = three years, 4 = four years, 5 = five years, and 6 = more than five years. *School attachment* was measured by the extent that adolescents agreed with the following statements “I feel close to people at this school,” “I feel like I am a part of this school,” and “I am happy to be at this school.” Items were rated using a 5-point Likert scale, with responses recoded such that higher values indicated greater school attachment (1 = strongly disagree to 5 = strongly agree). The sum of these items was used to create our measure of school attachment (α = 0.79). The school clubs adolescents could participate in were grouped into the following four club types: academic clubs, sports clubs, arts/music, and other clubs. *Academic clubs* represented the total number of scholastic-oriented clubs an adolescent participated in, including membership in the French club, German club, Latin club, Spanish club, history club, math club, science club, and honor society. *Sports clubs* represented the total number of athletic clubs an adolescent participated in, including membership in cheerleading, baseball, basketball, field hockey, football, ice hockey, soccer, swimming, tennis, track, volleyball, wresting, and other sports teams. *Arts/music clubs* represented the total number of arts related organizations an adolescent belonged to including the school band, chorus/choir, orchestra, and drama club. *Other clubs* represented the total number of vocational, civic, or other clubs/organizations an adolescent belonged to, including the book club, computer club, debate team, future farmers of America, newspaper club, student council, yearbook, and other clubs/organizations.

We also created several measures to characterize the school context at the school level. School sector type variables were generated such that all schools fit into one of the following school types: *Catholic schools*, *private schools* (includes non-Catholic religious affiliated or non-religious private schools), and public schools (includes comprehensive public schools, schools with open-enrollment/non-specialized curriculum, vocational/technical schools, and alternative schools; public schools served as the reference category). The school’s *dropout rate* was measured by summing the percent of students who dropped out of school across all measured grades (i.e., grade 7 through grade 12). For two schools, individual grade dropout percentages were unavailable so the total percent dropout rate was used instead.

Three measures were used to account for the physical distance between students and their school and students and their in-school peers. We had the latitude/longitude for each student (a synthetic version that displays relative position but not actual position, for obfuscation reasons); although we do not know the location of the school, we approximated it by computing the spatial centroid of all students in the school. This information was used to compute a measure of the *average distance to the school* for students in the school. A school-level measure of the *average distance to peers in school* was also constructed by determining the average distance between an adolescent and all other students in their school, and then computing the average of these values for all students in the school. To capture spatial clustering of adolescents, a measure for the *standard deviation of distance to peers in school* was created which is the standard deviation of the residential distance between students in the school.

We measured the adolescent’s personal network in the school. *Ties inside school* measured the total number of in-school friendship nominations made by a given adolescent (ego). *Ego in-degree* represents the number of times a given adolescent (ego) is nominated as a friend by other adolescents in their school. *Ego Bonacich centrality* measures the centrality or focal positioning of a given adolescent (ego) within a broader network using information on the centrality of the people he/she has nominated as friends (alters). *Personal network density* is the density of an adolescent’s personal network; the personal network is defined as persons whom ego named, or who named ego, as friends (i.e., the send-and-receive network).

We included measures addressing characteristics of the school network. The measure of *school network density* represents the relative density of the school network by dividing the observed density by the maximum possible density (i.e., the school’s density assuming all students have 10 ties within the school). *School network mutuality* captures the tendency for students in a school to reciprocate friendship nominations (i.e., mutuality index; [54]). The *size of school network* measures the total number of student questionnaires in the school. School network measures required a minimum response rate of 50%. In the few schools (*n* = 12) where this threshold was not met, values were imputed using available school/student data, except in the case of network size which was based on the total number of students on the school’s roster list.

We constructed measures of the neighborhood (block group) population context. *Population density* was measured using the metric of person/square kilometer. The *proportion of Black residents*, *Hispanic residents*, *Asian or Pacific Islander residents*, *and Other race residents* capture the racial/ethnic composition. *Racial heterogeneity* is measured as the dispersion of race composition. The *proportion of foreign born residents* under age 18 was also included. In the rare cases where block group estimates were missing, tract level or county level estimates were used instead.

We also added measures focused on the neighborhood (block group) social and structural context. Using principal components factor analysis, a factor variable was generated to measure *residential stability* based on the proportion occupied housing units moved into between 1985 and 1990 and proportion occupied housing units that were owner occupied at the block group level (α = 0.61). Block group-level *economic inequality* was similarly measured as a factor score variable but derived from a confirmatory factor analysis of the following four variables: standard deviation in the family income distribution, standard deviation in gross rent of renter-occupied housing units paying cash rent, standard deviation in home values of specified owner-occupied housing units, and standard deviation of household income. *Concentrated economic disadvantage* was also measured as a factor score variable derived from a confirmatory factor analysis of the proportion of people with income below the poverty level in 1989, proportion of homes with one a male or female heading the household and children under 18 years (single parent homes), the unemployment rate, and median household income all measured at the block group level. A confirmatory factor analysis of these two latent variables commonly used in the literature exhibited satisfactory approximate fit statistics (RMSEA = 0.098; CFI = 0.967).

Finally, we included several individual-level demographic variables as controls. These included measures of *female*, *grade level* (ranging from 6^th^ to 12^th^ grade), *native born* (or born in the U.S.), and race/ethnicity based on self-report: *Black*, *Latinx*, *Asian*/*Pacific Islander*, and *American Indian*/*Other*/*mixed race* (with Whites as the reference category). Race/ethnicity was coded to be mutually exclusive (e.g., Black represents those identifying as “Black” only). For any variables that were missing, we used the ‘ice’ command in Stata for multiple imputation of five datasets; this approach uses switching regressions, which is an iterative multivariable regression technique, to impute values.

### Analytic plan

A series of two-level multi-level models were estimated in Stata 14.1. One model included the number of ties outside the school as the outcome variable (mixed effects negative binomial regression), and the others included either past year adolescent deviance (mixed effects linear model) or alcohol use (mixed effects ordered logistic regression) as outcomes. The use of multi-level models was most appropriate given the nested structure of our data (students within schools) and multiple ecological contexts being explored. Multi-level models employing a three-level structure (students within block-groups within schools) had estimation problems on the full sample; when this model was tested on a subset of adolescents (saturated school sample; *N* = 20,745) from 123 schools, no significant differences were found across our three outcomes (number of ties outside the school, deviance, alcohol use) justifying the appropriateness of using a two-level modeling structure. All model fit statistics are reported in S1–S5 Tables.

## Results

### Out of school (non-school) friends

Individual, parental, contextual, and network variables (refer to Table 1 for sample descriptive statistics) were all examined to understand the factors underlying the endorsement of out of school friendships (see Table 2). Both parental measures were associated with the number of out of school friends, as adolescents with parents that are less supportive and engage in fewer monitoring behaviors report having more out of school friends. The incidence rate ratios (*IRR*) are presented in the right-most column. As shown, a one standard deviation increase in parental monitoring reduces the rate of out of school friends 5.3% (*IRR* = .947), while a similar increase in parental support reduces it by about 4% (*IRR* = .961). The combined effect of parental support and monitoring reduced the rate of out of school friends 9% (*IRR* = .911). On the other hand, adolescents with a more educated parent (primarily mother) reported more out of school ties (*IRR* = 1.027).

**Table 2:** Predicting ties outside the school based on characteristics of individual, parents, school, neighborhood, and network (*N* = 81, 674).

	Coef	z-value		IRR
Ties inside school	-0.200	-(35.19)	**	0.819
***Parental measures***				
Parental monitoring	-0.465	-(8.69)	**	0.947
Parental support	-0.143	-(6.81)	**	0.961
Education (mother)	0.027	(5.50)	**	1.027
***School clubs measures***				
Number of academic clubs	0.003	(0.35)		1.003
Number of sports clubs	0.004	(0.84)		1.004
Number of arts clubs	0.055	(5.15)	**	1.057
Number of other clubs	0.063	(7.77)	**	1.065
***School level variables***				
School dropout rate	-0.005	-(2.15)	*	0.934
Catholic school	0.536	(3.83)	**	1.709
Private school	0.271	(1.71)	†	1.311
Average distance to school^a^	0.237	(1.48)		1.481
Standard deviation of distance between students in school	0.000	(0.99)		1.039
Average distance between students in school^a^	-0.188	-(1.53)		0.648
***School network measures***				
Density	0.065	(0.21)		1.006
Mutuality index	0.619	(0.98)		1.029
Size of school (in 1,000s)	-0.167	-(3.34)	**	0.887
***Personal network measures***				
In-degree	0.003	(1.91)	†	1.014
Bonacich centrality	0.111	(4.32)	**	1.074
Personal network density	-0.050	-(1.13)		0.992
***Block group level variables***				
Economic inequality^b^	-0.005	-(5.45)	**	0.962
Concentrated disadvantage	-0.192	-(2.81)	**	0.978
Residential stability	0.037	(5.43)	**	1.037
Population density	0.017	(8.51)	**	1.057
Proportion Black	0.020	(1.52)		1.009
Proportion Latinx	-0.016	-(1.02)		0.993
Proportion Asian	-0.055	-(4.24)	**	0.974
Proportion Other	-0.009	-(0.62)		0.996
Racial/ethnic heterogeneity	0.013	(0.72)		1.005
Proportion foreign born	-0.020	-(1.46)		0.990
***Individual level variables***				
Female	0.465	(38.44)	**	1.591
Grade	0.126	(18.44)	**	1.134
Black	-0.166	-(7.93)	**	0.847
Latinx	-0.236	-(7.03)	**	0.789
Asian	-0.080	-(2.46)	*	0.923
Native American/Other/Mixed Race	-0.074	-(4.31)	**	0.928
Native born	0.187	(8.29)	**	1.206
Years in this school	-0.073	-(12.62)	**	0.929
Intercept	-0.697	-(3.07)	**	

*Note*. Multi-level negative binomial regression model estimated on adolescents within 126 schools, *z*-score values presented in parenthesis. IRR values are incidence rate ratios, which are computed by exponentiating the coefficient for dichotomous variables, or exponentiating the coefficient multiplied by the standard deviation of the variable for continuous variables.

^a^Average distance to school and average distance between students in school measures rescaled (divided by 100,000)

^b^Economic inequality coefficient value rescaled (multiplied by 1,000)

†*p* < .10.

* p < .05.

** *p* < .01.

The school context also is associated with out of school tie formation. Participation in an additional art club is associated with more out of school ties (*IRR* = 1.057) as is participation in an additional “other” club (student council, yearbook, etc.; *IRR* = 1.065). Aggregating across all club variables, an adolescent’s rate of out of school ties increased 13.4% (*IRR* = 1.134) if they were involved in all four club types. As expected, students who have been in the school longer have fewer out of school ties: each additional year in the school reduces the rate of out of school ties 7%. On average, a student in a Catholic school has 70% more out of school ties than a public school student (*IRR* = 1.709). A student in a school with a 15 percentage point higher dropout rate (one standard deviation) has a about 7% fewer out of school ties. For Catholic schools with a low dropout rate, students have 83% (*IRR* = 1.831) more out of school ties than those in public schools with an average dropout rate.

A student’s position in the school network is related to their number of out of school ties. A student with higher Bonacich centrality (one standard deviation) has 7% more out of school ties (*IRR* = 1.074). Students in larger schools also reported 9% fewer out of school ties (*IRR* = .887).

Regarding the neighborhood context, all three structural block group measures are significantly related to the number of out of school ties. Adolescents living in high inequality (*IRR* = .962) or high concentrated disadvantage (*IRR* = .978) neighborhoods have fewer out of school ties, whereas those in high residentially stable neighborhoods have more out of school ties (*IRR* = 1.037), although these are small effects. The combined effect of living in a neighborhood with high inequality and disadvantage, and low residential stability, results in 9% fewer out of school ties (*IRR* = 0.907). A one standard deviation increase in the Asian and Pacific Islander population reduced the rate of out of school ties by approximately 3% (*IRR* = .974).

In terms of individual-level control variables, adolescents with fewer in-school friendships, females, adolescents in higher grades, and those born in the United States have more out of school friends. All non-White adolescents (i.e., Black, Latinx, Asian/Pacific Islander, Native American/other/mixed race) reported fewer out of school friends when compared to White adolescents.

### Adolescent deviance and alcohol use

Models with deviance or alcohol use as outcomes are presented in Table 3. First, we estimated a model which does not include our ties outside of school measure, and then we estimated a model including this variable, to assess how much the results change when accounting for out of school friendships. In Eq 1, we observe that the presence of more in-school ties is associated with modestly lower levels of deviance (β = -.002). In Eq 2, in which we include the count of ties outside the school, the relationship of in-school ties with deviance becomes nonsignificant. In contrast, the presence of more out of school ties is associated with higher levels of deviance (β = .021). Having one additional tie inside the school and one more outside the school increased deviance by .022.

**Table 3:** Predicting deviance and alcohol use with social ties inside and outside the school (*N* = 81, 674).

	Deviance			Deviance			Alcohol use			Alcohol use		
	Coef.		Beta	Coef.		Beta	Coef.		OR	Coef.		OR
Ties inside school	-0.001	†	-0.002	0.000		0.001	0.015	**	1.015	0.026	**	1.027
	-(1.89)			(0.99)			(6.17)			(10.64)		
Ties outside school				0.007	**	0.021				0.066	**	1.068
				(12.12)						(19.35)		
***Parental measures***												
Parental monitoring	-0.145	**	-0.052	-0.140	**	-0.051	-1.515	**	0.838	-1.471	**	0.843
	-(14.51)			-(14.04)			-(24.11)			-(23.39)		
Parental support	-0.184	**	-0.157	-0.183	**	-0.156	-0.669	**	0.832	-0.662	**	0.833
	-(46.41)			-(46.23)			-(27.00)			-(26.72)		
***School level variables***												
School dropout rate	0.001	**	0.068	0.002	**	0.072	0.008	**	1.132	0.009	**	1.147
	(4.50)			(4.71)			(3.52)			(3.85)		
***Block group level variables***												
Concentrated disadvantage	0.067	**	0.024	0.068	**	0.025	0.241	**	1.029	0.255	**	1.031
	(6.14)			(6.29)			(3.54)			(3.75)		
***Individual level variables***												
Female	-0.075	**	-0.232	-0.079	**	-0.244	-0.308	**	0.735	-0.348	**	0.706
	-(34.59)			-(36.03)			-(22.67)			-(25.27)		
Grade	-0.006	**	-0.018	-0.007	**	-0.021	0.184	**	1.202	0.174	**	1.190
	-(4.67)			-(5.52)			(23.08)			(21.67)		
Black	-0.026	**	-0.079	-0.024	**	-0.076	-0.361	**	0.697	-0.352	**	0.704
	-(6.65)			-(6.36)			-(14.90)			-(14.49)		
Latinx	0.029	**	0.091	0.031	**	0.097	0.117	**	1.124	0.140	**	1.150
	(4.80)			(5.15)			(3.07)			(3.67)		
Asian	-0.047	**	-0.023	-0.046	**	-0.022	-0.755	**	0.470	-0.749	**	0.473
	-(7.81)			-(7.67)			-(18.56)			-(18.37)		
Native American, Other, or Mixed Race	0.038	**	0.117	0.039	**	0.120	0.130	**	1.139	0.137	**	1.146
	(12.04)			(12.26)			(6.60)			(6.95)		
Native born	0.026	**	0.082	0.025	**	0.076	0.304	**	1.355	0.287	**	1.333
	(6.29)			(5.90)			(11.18)			(10.55)		
School Attachment	-0.021	**	-0.197	-0.020	**	-0.194	-0.086	**	0.769	-0.083	**	0.776
	-(57.47)			-(56.55)			-(37.49)			-(36.11)		
Years in this school	0.014	**	0.044	0.015	**	0.046	0.052	**	1.054	0.060	**	1.062
	(13.34)			(14.00)			(7.63)			(8.74)		
Intercept	0.224	**		0.217	**							
	(17.72)			(17.14)								
Cutpoint 1							1.051	**		1.120	**	
							(12.70)			(13.48)		
Cutpoint 2							2.232	**		2.307	**	
							(26.86)			(27.62)		
Cutpoint 3							2.810	**		2.886	**	
							(33.71)			(34.45)		
Cutpoint 4							3.562	**		3.641	**	
							(42.54)			(43.26)		
Cutpoint 5							4.695	**		4.775	**	
							(55.31)			(55.97)		
Cutpoint 6							5.446	**		5.526	**	
							(62.84)			(63.47)		

*Note*. Multi-level models estimated on adolescents within 126 schools, *z*-score values presented in parenthesis. Deviance estimated using a mixed effects negative binomial regression. Alcohol use estimated using a mixed effects ordered logistic regression. OR values are odds ratios, which are computed by exponentiating the coefficient for dichotomous variables, or exponentiating the coefficient multiplied by the standard deviation of the variable for continuous variables. BETA values capture the change in standard deviations of deviance for a one unit change in dichotomous variables, or a one standard deviation change in continuous variables. For ties inside and outside of school, BETA and OR values represent a change in the standard deviation or odds of deviance or alcohol use given a one unit change in the predictor variables (i.e., the addition of a tie).

†*p* < .10.

* *p* < .05.

** *p <* .01.

Next, we turn to models with alcohol use as the outcome variable (see the right side of Table 3). The initial positive relationship between in-school ties and alcohol use in column 3 (OR = 1.015) is strengthened after adjusting for out of school ties in column 4 (OR = 1.027). In addition, there is a notable positive relationship between out of school ties and alcohol use (OR = 1.068). Furthermore, having one more tie inside the school and one more outside of school increases the odds for a one unit increase in alcohol use by 9.6%.

The other variables in these models generally behave as expected. Adolescents with more parental monitoring or support engage in less deviance or alcohol use, as do adolescents in schools with lower dropout rates. Combining our parental variables, we note that a one standard deviation increase in both parental monitoring and support decreased adolescent deviance by .207 and reduced the odds of a one unit increase in alcohol use by 30%. Living in neighborhoods with higher concentrated disadvantage is associated with higher levels of deviance or alcohol use. Males, native born youth, and those with more years in the school engage in higher levels of deviance or alcohol use, on average. Adolescents in higher grades engage in more alcohol use but less deviance. Latinx and Native American/other/mixed race adolescents engage in more deviance and alcohol use compared to White adolescents, on average, while the opposite relationship is noted for Black and Asian adolescents. Adolescents with strong school bonds or attachment also engage in less deviance and alcohol use.

#### Moderating effects: Context impact on ties

Finally, we wished to assess whether the particular neighborhood or school context impacted some of these tie formation patterns. First, it is possible that an adolescent will have more out of school ties if their own race/ethnicity matched that of the majority of residents in their own neighborhood. We assessed this in ancillary models by testing the interaction of neighborhood and personal racial/ethnic identity (e.g., Black or African American identity x neighborhood proportion of Black or African Americans). There were no significant interaction effects detected in these ancillary analyses. However, the limited diversity of our sample of students (e.g., less than 5% Latinx and Asian or Pacific Islander) may have contributed to these null findings.

Second, we wished to assess whether the population differences between public schools and non-public schools in terms of student diversity [36] have important consequences. We assessed this in ancillary models in which we examined the interaction effect of school type and minority status for out of school ties. We anticipated that non-White students in private schools would have more out of school ties given that they may be likely to be relatively racially isolated in private schools. However, this was not the case as we found essentially no difference in the number of out of school friends endorsed by White and non-White students in private and Catholic schools. On the other hand, in public schools we found that White students reported more out of school friends than their non-White counterparts (see S6 Table and S1 Fig). This may occur if White students in public schools have fewer opportunities to form “same-ethnic” friendships (compared to white students in private schools) and thus may look outside the school for such peers [37]. Indeed, in our sample, public schools were 47% non-White whereas this value was 24% in private schools.

## Discussion

The present study involved a multi-contextual examination of adolescent out of school friendships and their impact on risky health behaviors. Even though it is well-known that adolescents have friends within and outside the school, financial constraints and study feasibility have often prohibited scholars from examining out of school friendships on a large scale. Using the Add Health dataset, here we were able to begin to look at the individual, parental, social, and contextual characteristics that contribute to having out of school friends. Additionally, we also examined the factors that promote adolescent deviance and alcohol use, one of which was a higher number of out of school friends. To answer our first study question: “Who has out of school friends?” we studied an array of important social or structural contexts that likely influence adolescent tie formation, in addition to the adolescent’s own attributes. This included the familial context approximated by the parent-child relationship, the school context, the school/personal network context, and the neighborhood context. To answer the second study question: “What is the significance of out of school ties on adolescent deviance and alcohol use?” we estimated a more parsimonious model including only established theoretically significant predictors (parental support and monitoring, neighborhood disadvantage, school drop-out rate etc.) along with in-school and out of school tie measures. We found that different contexts exhibited varying importance in understanding the number of out of school ties that adolescents have, as well as their tendency to be engage in a deviant manner or drink alcohol.

Findings suggest that our two measures of parent-child relationships have consequences for out of school ties, deviance, and alcohol use. Adolescents who indicate greater support or monitoring from their parents had fewer out of school ties and reported less deviance or alcohol use. As supportive and high monitoring parents tend to be aware of their child’s friends and activities, it is no surprise that adolescents with involved and caring parents partake in fewer deviant activities and drink less [29]. In addition, the more educated the parent the more likely the adolescent is to have out of school friendships. As higher educational attainment often corresponds to increased wealth, this finding may be a result of more educated parents having the financial means to expose their children to out of school peers via summer camps, private sports teams, and other paid social or educational opportunities. There was also evidence that adolescent activity fostered by the school was related to the number of out of school ties albeit not in the expected direction. Adolescents who belonged to more arts clubs and other clubs (student council, computer club, newspaper club etc.) reported more out of school ties. One explanation for this association could be found in the fact that though U.S. school clubs typically hold regular meetings on site, students enrolled in clubs often also have multiple opportunities to compete against students from other schools (debate team competition, meets or play-offs, etc.). Clubs thus provide youth with increased contact to a diverse set of peers with whom they have shared interests, leading to the development of cross-race and age friendships [55], along with potentially interschool or cross-school friendships.

As predicted, adolescents who spent more years in a given school were less likely to report out of school friendships. Though we had hypothesized that more years in school would encourage adolescents to engage in prosocial behaviors, our results tell a different story. Deviance and alcohol use increase for more seasoned students, controlling for grade level and school attachment. This result may be a consequence of the age-crime curve [56, 57], or the fact that deviant acts (including alcohol law violations) increase from late childhood to middle or late adolescence, after which they decline. Adolescents in the same school for a longer period of time are thus more likely to fall into the peak age range for offending which could explain the associations between years in school and self-reported deviance/alcohol use.

Students enrolled in Catholic schools endorse more out of school friends which may be a consequence of their school not being located near their home. Students are more likely to befriend peers who live near them or near another friend (spatial propinquity; [58]). For non-public school students, distance and transportation constraints could results in individuals reporting more out of school friendships than in-school friends. Adolescents in schools with high dropout rates reported fewer out of school friends than students in schools with lower dropout rates. This unexpected finding may be because, in schools with high dropout rates, enrolled students have more in common (daily experiences, ambitions, etc.) with schoolmates than out of school peers. Reciprocated in-school friendships have also been shown to improve academic outcomes and decrease dropout propensity [59]. The absence of out of school friends might therefore be a protective strategy for adolescents in high dropout school environments–helping them resist the temptation to dropout themselves. As expected, students in schools with high dropout rates engaged in more deviant behaviors and alcohol use compared to students in low dropout schools. Poor school environments have been previously associated with delinquency engagement [60]. For students in high dropout schools, rule-breaking behavior and occasional substance use may be viewed as more permissible, resulting in higher self-reported deviance and alcohol use.

Regarding the school network, adolescents who were central to the network were more likely to befriend someone outside of their school. One explanation for this surprising finding is that central adolescents are in an ideal position within the network to access more non-school peers through mutual connections (i.e., becoming friends with their friend’s friend; [49]) which may allow for more out of school friendships. Adolescents in larger school networks on the other hand reported few out of school ties. Friendships are often forged between youth with similar interests or backgrounds [37, 55], so it makes sense that the larger the school network, the less adolescents may need to or want to venture outside of school for friends.

Neighborhood residential stability was related to more out of school friends whereas neighborhood disadvantage and inequality were related to fewer out of school friends, although these were relatively small effects. That is, the more established the neighborhood, the more likely adolescents are to befriend non-school persons, whereas living in an area compounded by multiple forms of disadvantage (poverty, unemployment, etc.) or residential inequity is less likely to produce non-school friendships. Disadvantaged neighborhoods are often characterized as unsafe crime-ridden areas [61] therefore adolescents and their parents may prefer friendships to blossom in school rather than outside of the school. Supplemental analyses were conducted to see if parental monitoring moderated the association between neighborhood inequality/disadvantage and out of school friendship ties. Our results (see S7 and S8 Tables) indicated that adolescents residing in neighborhoods high in economic inequality or concentrated disadvantage reported few out of school friendships, with the lowest number of ties reported by those who also had high monitoring parents. This suggests that although the neighborhood context can provide adolescents with opportunities to befriend non-school peers, parental restrictions can ultimately curtail out of school friendship formation. Adolescents living in neighborhoods that were disadvantaged were also found to be more deviant and substance using than adolescents from more prosperous neighborhoods. There was little evidence that the block-group racial/ethnic context mattered for out of school friends. Replication efforts on more diverse student populations are recommended.

Friendships had different effects on adolescent deviance and alcohol use depending on whether they were in-school or out of school ties. For deviant behaviors, more in-school friends were initially associated with less deviance but after out of school friends were included, only out of school friends were associated with greater deviance. For alcohol use, when both types of ties were present, out of school friendship ties were a more robust predictor of adolescent alcohol use behavior than in-school ties. Though statistically significant, the effect from out of school ties on deviance/alcohol use was small when compared to effect of parenting measures and other individual level predictors (e.g., gender, native born, and school attachment). Still, consistent with prior literature [3, 16, 18], these findings suggest that out of school friendships are more likely to promote risky or problematic behaviors than in-school friendships. One reason for this may have to do with adolescent peer acceptance and school attachment. As noted in the present study, adolescents who report greater levels of school attachment report lower levels of deviance/alcohol use. Evidence by Kiesner and colleagues [1, 2] finds that adolescents who are more antisocial experience lower classroom peer acceptance, tend to befriend others outside of the school setting (e.g., in their neighborhood), and spend time socializing with their friends only after school. This implies that adolescents with more out of school friendships, differ from youth with more in school friendships, in terms of their opportunity to socialize in unstructured settings ripe for acts of deviance [17, 21, 33] and in their tendency to be socially rejected for engaging in rule-breaking behavior [2]. Additional research is needed on out of school friends to more clearly articulate how these friends differ from the more well-studied school friends. Though it may be tempting to assume out of school friends are “worse” than in-school friends–to do so would be inaccurate and an oversimplification. Adolescent friendships are always best evaluated with an understanding of the individual and their available resources at home, in school, and in their neighborhood [60]. As discussed early on by Brazil (2016) and expanded upon by Gaias and colleagues [63], more studies should also examine the “proximal process” or the interactions between adolescents and their environments including *both* school and neighborhood attributes as both have important implications for adolescent development [62, 63]. Our supplemental analyses (S6–S8 Tables) began to consider such proximal processes on out of school friendship ties while simultaneously adjusting for school and neighborhood factors, but more work is needed in this area. Future interventions aimed at reducing adolescent deviance and alcohol use should therefore consider a more holistic approach that involves collaborations with school, community, and family stakeholders to decrease student dropout rates, encourage school attachment, improve the socio-economic status of student neighborhoods, educate parents on positive parenting practices, and promote healthy peer friendships.

This study has some limitations. The main limitation was the lack of information describing out of school friends and number of friendship ties adolescents could list. Future network studies should consider collecting information on out of school friends (e.g., age, ethnicity, and residential details) and where friendship ties form. Gathering these data, among others, would help illuminate who these friends are with greater certainty. Also, instead of restricting the number of friendship nominations to 10 friends (5 male, 5 female), future network studies are encouraged to allow adolescents to list as many in school and out of school friends as they want, as doing so would showcase the complete network profile of adolescents. A second limitation relates to the diversity of the school community. Future efforts to replicate our work on a more diverse student sample is recommended to get at how personal race/ethnicity interacts with the demographics of the school and neighborhood. Finally, these data are over 20 years old. The advent and popularity of social network sites like Facebook or Twitter, websites like meetup.com, and cellphones mean friendships inside and outside of school are more accessible than ever for adolescents. How these modern-day friendships inform deviance and alcohol use therefore remains an open question for scholars.

## Conclusions

An ecological approach was used in this study to assess deviance, alcohol use, and friendship formation beyond the often measured school environment. Our intentional choice to examine the multiple contexts that bear on out of school social ties was predicated on the fact that adolescents are embedded in a fluid, ever-changing system. How youth navigate through their complex social world matters–as deviancy and friendships arise when the right mix of people, opportunity, and place converge. This means that instead of presuming that different types of friendships are prosocial or antisocial, an effort must be made to examine these relationships *in context*. Our results indicate that out of school friendships, above and beyond in-school friendships, are important for adolescent deviance and alcohol use, and that youth with out of school friends are not completely disengaged from their schools (e.g., they participate in school clubs). Having identified several leverage points–out of school friends, parents, the school network, and the neighborhood–our findings can support scholars and policy makers interested in reducing deviance and underage drinking. During adolescence, youth spend a lot of their time forming and maintaining their friendships. Only recently have scholars begin to explore non-traditional friendships made online or outside the classroom. By including this “missed” source of friendship influence, along with the broader ecological context, scholars will be better able to accurately understand the risk and protective factors associated with adolescent deviance and alcohol use.

## Supporting information

S1 Table Results from MLM predicting out of school friendships. (DOCX)

S2 Table Results from MLM predicting alcohol use (no out of school ties). (DOCX)

S3 Table Results from MLM predicting alcohol use with out of school ties. (DOCX)

S4 Table Results from MLM predicting deviance (no out of school ties). (DOCX)

S5 Table Results from MLM predicting deviance with out of school ties. (DOCX)

S6 Table Results from MLM predicting out of school friendships with public school type x White race. (DOCX)

S7 Table Results from MLM predicting out of school friendships with parental monitoring x neighborhood inequality. (DOCX)

S8 Table Results from MLM predicting out of school friendships with parental monitoring x neighborhood disadvantage. (DOCX)

S1 Fig Public school type x White race interaction. (PDF)

## References

[1] Kiesner J, Kerr M, Stattin H. “Very important persons” in adolescence: Going beyond in-school, single friendships in the study of peer homophily. J Adolesc. 2004;27(5):545–60. 10.1016/j.adolescence.2004.06.007 15475046

[2] Kiesner J, Poulin F, Nicotra E. Peer relations across contexts: Individual-network homophily and network inclusion in and after school. Child Dev. 2003;74(5):1328–43. 10.1111/1467-8624.00610 14552401

[3] Ennett ST, Bauman KE, Hussong A, Faris R, Foshee VA, Cai L, et al The peer context of adolescent substance use: Findings from social network analysis. J Res Adolesc. 2006;16(2):159–86.

[4] Ennett ST, Foshee VA, Bauman KE, Hussong A, Cai L, Luz H, et al The social ecology of adolescent alcohol misuse. Child Dev. 2008;79(6):1777–91. 10.1111/j.1467-8624.2008.01225.x 19037949 PMC2597371

[5] Dishion TJ, Andrews DW, Crosby L. Antisocial boys and their friends in early adolescence: Relationship characteristics, quality, and interactional process. Child Dev. 1995;66(1):139–51. 10.1111/j.1467-8624.1995.tb00861.x 7497821

[6] Haynie DL. Friendship networks and delinquency: The relative nature of peer delinquency. Journal of Quantitative Criminology. 2002;18(2):99–134.

[7] Piquero NL, Gover AR, MacDonald JM, Piquero AR. The influence of delinquent peers on delinquency: Does gender matter? Youth Soc. 2005;36(3):251–75.

[8] Scholte RHJ, Poelen EAP, Willemsen G, Boomsma DI, Engels RCME. Relative risks of adolescent and young adult alcohol use: The role of drinking fathers, mothers, siblings, and friends. Addict Behav. 2008;33(1):1–14. 10.1016/j.addbeh.2007.04.015 17490824

[9] Ripley RM, Snijders TAB, Boda Z, Voros A, Preciado P. Manual for RSIENA. Oxford: University of Oxford; 2016.

[10] Knecht AB. Friendship selection and friends’ influence: Dynamics of networks and actor attributes in early adolescence. University of Utrecht Utrecht, The Netherlands; 2008.

[11] Haynie DL, Doogan NJ, Soller B. Gender, friendship networks, and delinquency: A dynamic network approach. Criminology. 2014;52(4):688–722. 10.1111/1745-9125.12052 26097241 PMC4469075

[12] Wang C, Hipp JR, Butts CT, Jose R, Lakon CM. Peer influence, peer selection and adolescent alcohol use: A simulation study using a dynamic network model of friendship ties and alcohol use. Prev Sci. 2017;18(4):382–93. 10.1007/s11121-017-0773-5 28361198 PMC10950262

[13] Wang C, Hipp JR, Butts CT, Jose R, Lakon CM. Alcohol use among adolescent youth: The role of friendship networks and family factors in multiple school studies. PLoS One. 2015;10(3):1–19. 10.1371/journal.pone.0119965 25756364 PMC4355410

[14] Osgood DW, Ragan DT, Wallace L, Gest SD, Feinberg ME, Moody J. Peers and the emergence of alcohol use: Influence and selection processes in adolescent friendship networks. Journal of Research on Adolescence. 2013;23(3):500–512. 10.1111/jora.12059 24307830 PMC3844135

[15] Burk WJ, Steglich CEG, Snijders TAB. Beyond dyadic interdependence: Actor-oriented models for co-evolving social networks and individual behaviors. Int J Behav Dev. 2007;31(4):397–404.

[16] Jose R, Hipp JR, Butts CT, Wang C, Lakon CM. Network structure, influence, selection, and adolescent delinquent behavior: Unpacking a dynamic process. Crim Justice Behav. 2016;43(2):264–84.

[17] Weerman FM. Delinquent peers in context: A longitudinal network analysis of selection and influence effects. Criminology. 2011;49(1):253–86.

[18] Kiesner J, Poulin F, Dishion TJ. Adolescent substance use with friends: Moderating and mediating effects of parental monitoring and peer activity. Merrill-Palmer Quarterly (Wayne State Univ Press). 2010;56(4):529–56. 10.1353/mpq.2010.0002 21165170 PMC3002110

[19] Tucker JS, Pollard MS, de la Haye K, Kennedy DP, Green HD. Neighborhood characteristics and the initiation of marijuana use and binge drinking. Drug Alcohol Depend. 2013;128(1–2):83–9. 10.1016/j.drugalcdep.2012.08.006 22938829 PMC3521064

[20] Osgood DW, Wilson JK, O’Malley PM, Bachman JG, Johnston LD. Routine activities and individual deviant behavior. Am Sociol Rev. 1996;61(4):635–55.

[21] Hoeben EM, Osgood DW, Siennick SE, Weerman FM. Hanging out with the wrong crowd? The role of unstructured socializing in adolescents’ specialization in delinquency and substance use. Journal of Quantitative Criminology. 2020 [Advance Online Publication]. Available from: 10.1007/s10940-019-09419-8 32863562 PMC7453844

[22] Bowlby J. A secure base: Parent-child attachmentand healthy human development. New York: Basic Books; 1988.

[23] Bowlby J. Attachment. New York: Basic Books; 2008.

[24] Hoeve M, Dubas JS, Eichelsheim VI, van der Laan PH, Smeenk W, Gerris JRM. The relationship between parenting and delinquency: A meta-analysis. J Abnorm Child Psychol. 2009;37(6):749–75. 10.1007/s10802-009-9310-8 19263213 PMC2708328

[25] Ryan SM, Jorm AF, Lubman DI. Parenting factors associated with reduced adolescent alcohol use: A systematic review of longitudinal studies. Aust N Z J Psychiatry. 2010;44(9):774–83. 10.1080/00048674.2010.501759 20815663

[26] Lakon CM, Wang C, Butts CT, Jose R, Timberlake DS, Hipp JR. A dynamic model of adolescent friendship networks, parental influences, and smoking. J Youth Adolesc. 2015;44(9):1767–86. 10.1007/s10964-014-0187-7 25239115 PMC4397181

[27] Parker JS, Benson MJ. Parent-adolescent relations and adolescent functioning: Self-esteem, substance abuse, and delinquency. Adolescence. 2004;39(155):519–30. 15673227

[28] Gault-Sherman M. It’s a two-way street: The bidirectional relationship between parenting and delinquency. J Youth Adolesc. 2012;41(2):121–45. 10.1007/s10964-011-9656-4 21431892

[29] Donaldson CD, Handren LM, Crano WD. The enduring impact of parents’ monitoring, warmth, expectancies, and alcohol use on their children’s future binge drinking and arrests: A longitudinal analysis. Prev Sci. 2016;17(5):606–14. 10.1007/s11121-016-0656-1 27178008 PMC5901752

[30] Deutsch AR, Crockett LJ, Wolff JM, Russell ST. Parent and peer pathways to adolescent delinquency: Variations by ethnicity and neighborhood context. J Youth Adolesc. 2012;41(8):1078–94. 10.1007/s10964-012-9754-y 22460729

[31] Marshal MP, Chassin L. Peer influence on adolescent alcohol use: The moderating role of parental support and discipline. Appl Dev Sci. 2000;4(2):80–8.

[32] Barnes GM, Hoffman JH, Welte JW, Farrell MP, Dintcheff BA. Effects of parental monitoring and peer deviance on substance use and delinquency. J Marriage Fam. 2006;68(4):1084–104.

[33] Osgood DW, Anderson AL. Unstructured socializing and rates of delinquency. Criminology. 2004;42(3):519–49.

[34] Tilton-Weaver LC, Burk WJ, Kerr M, Stattin H. Can parental monitoring and peer management reduce the selection or influence of delinquent peers? Testing the question using a dynamic social network approach. Dev Psychol. 2013;49(11):2057–70. 10.1037/a0031854 23421802

[35] Mahoney JL, Larson RW, Eccles JS, editors. Organized activities as contexts of development: Extracurricular activities, after-school and community programs. Mahwah, NJ: Lawrence Erlbaum and Associates; 2005.

[36] U.S. Department of Education, National Center for Education Statistics. Findings from the Condition of Education 1997: Public and private schools: How do they differ?. 1997. Available from: https://nces.ed.gov/pubs97/97983.pdf

[37] Munniksma A, Scheepers P, Stark TH, Tolsma J. The impact of adolescents’ classroom and neighborhood ethnic diversity on same- and cross-ethnic friendships within classrooms. J Res Adolesc. 2016;27(1):20–33. 10.1111/jora.12248 28498532

[38] Rhodes A. The age of belonging: Friendship formation after residential mobility. Soc Forces. 2018;97(2):583–606.

[39] Jose PE, Ryan N, Pryor J. Does social connectedness promote a greater sense of well-being in adolescence over time? J Res Adolesc. 2012;22(2):235–51.

[40] Henry KL, Knight KE, Thornberry TP. School disengagement as a predictor of dropout, delinquency, and problem substance use during adolescence and early adulthood. J Youth Adolesc. 2012;41(2):156–66. 10.1007/s10964-011-9665-3 21523389 PMC4516271

[41] Monahan KC, Oesterle S, Hawkins JD. Predictors and consequences of school connectedness: The case for prevention. The Prevention Researcher. 2010;17(3):3–6.

[42] Roebroek L, Koning IM. The reciprocal relation between adolescents’ school engagement and alcohol consumption, and the role of parental support. Prev Sci. 2016;17(2):218–26. 10.1007/s11121-015-0598-z 26334710 PMC4718970

[43] Hartup WW. Adolescents and their friends. New Dir Child Dev. 1993;60:3–22. 10.1002/cd.23219936003 8414122

[44] Preciado P, Snijders TAB, Burk WJ, Stattin H, Kerr M. Does proximity matter? Distance dependence of adolescent friendships. Soc Networks. 2012;34(1):18–31. 10.1016/j.socnet.2011.01.002 25530664 PMC4268773

[45] Bond L, Lusher D, Williams I, Butler H. Friends or foes? Relational dissonance and adolescent psychological wellbeing. PLOS ONE. 2014;9(2). 10.1371/journal.pone.0083388 24498257 PMC3911895

[46] McPherson M, Smith-Lovin L, Cook JM. Birds of a feather: Homophily in social networks. Annu Rev Sociol. 2001;27:415–44.

[47] Currarini S, Jackson MO, Pin P. Identifying the roles of race-based choice and chance in high school friendship network formation. Proc Natl Acad Sci. 2010;107(11):4857–61. 10.1073/pnas.0911793107 20212129 PMC2841897

[48] Mouw T, Entwisle B. Residential segregation and interracial friendship in schools. Am J Sociol. 2006;112(2):394–441.

[49] Goodreau SM, Kitts JA, Morris M. Birds of a feather, or friend of a friend? Using exponential random graph models to investigate adolescent social networks. Demography. 2009;46(1):103–25. 10.1353/dem.0.0045 19348111 PMC2831261

[50] Guest AM, Cover JK, Matsueda RL, Kubrin CE. Neighborhood context and neighboring ties. City Community. 2006;5(4):363–85.

[51] Wikström P-OH, Loeber R. Do disadvantaged neighborhoods cause well-adjusted children to become adolescent delinquents? A study of male juvenile serious offending, individual risk and protective factors, and neighborhood context. Criminology. 2000;38(4):1109–42.

[52] Leventhal T, Brooks-Gunn J. The neighborhoods they live in: The effects of neighborhood residence on child and adolescent outcomes. Psychol Bull. 2000;126(2):309–37. 10.1037/0033-2909.126.2.309 10748645

[53] Augustyn MB, McGloin JM. The risk of informal socializing with peers: Considering gender differences across predatory delinquency and substance use. Justice Q. 2013;30(1):117–43.

[54] Katz L, Powell JH. Measurement of the tendency toward reciprocation of choice. Sociometry. 1955;18(4):403–9.

[55] Fredricks JA, Simpkins SD. Organized out-of-school activities and peer relationships: Theoretical perspectives and previous research. New Dir Child Adolesc Dev. 2013;2013(140):1–17. 10.1002/cad.20034 23766093

[56] Farrington DP. Age and crime. Crime & Justice. 1986;7:189–205.

[57] Loeber R, Menting B, Lynam DR, Moffitt TE, Stouthamer-Loeber M, Stallings R, et al Findings from the Pittsburgh youth study: Cognitive impulsivity and intelligence as predictors of the age-crime curve. J Am Acad Child Adolesc Psychiatry. 2012;51(11):1136–49. 10.1016/j.jaac.2012.08.019 23101740

[58] Kruse H, Smith S, van Tubergen F, Maas I. From neighbors to school friends? How adolescents’ place of residence relates to same-ethnic school friendships. Soc Networks. 2016;44:130–42.

[59] Ricard NC, Pelletier LG. Dropping out of high school: The role of parent and teacher self-determination support, reciprocal friendships and academic motivation. Contemp Educ Psychol. 2016;44–45:32–40.

[60] Fine A, Mahler A, Steinberg L, Frick PJ, Cauffman E. Individual in context: The role of impulse control on the association between the home, school, and neighborhood developmental contexts and adolescent delinquency. J Youth Adolesc. 2017;46(7):1488–502. 10.1007/s10964-016-0565-4 27663574

[61] Sampson RJ, Groves WB. Community structure and crime: Testing social-disorganization theory. Am J Sociol. 1989;94(4):774–802.

[62] Brazil N. The effects of social context on youth outcomes: Studying neighborhoods and schools simultaneously. Teach Coll Rec. 2016;118(7):1–30.

[63] Gaias LM, Lindstrom Johnson S, White RMB, Pettigrew J, Dumka L. Understanding school–neighborhood mesosystemic effects on adolescent development. Adolesc Res Rev. 2018;3(3):301–19.

