# Supplementary material for: A multi-contextual examination of non-school friendships and their impact on adolescent deviance and alcohol use
Source: PLoS One. 2021 Feb 10;16(2):e0245837. doi: 10.1371/journal.pone.0245837 (PMC7875427; doi:10.1371/journal.pone.0245837)
Supplement: S1 Table — (DOCX) [file pone.0245837.s001.docx]

| **S1 Table. Results from MLM predicting out of school friendships** | | | | | |  | |  |
| --- | --- | --- | --- | --- | --- | --- | --- | --- |
|  |  | Estimate | Standard Error | *z* | *p* | 95% *CI* | | |
| Ties inside school | | -0.200 | 0.006 | -35.19 | 0.000 | -0.211 | -0.188 | |
| ***Parental measures*** | |  |  |  |  |  |  | |
| Parental monitoring | | -0.465 | 0.054 | -8.69 | 0.000 | -0.570 | -0.360 | |
| Parental support | | -0.143 | 0.021 | -6.81 | 0.000 | -0.184 | -0.102 | |
| Education (mother) | | 0.027 | 0.005 | 5.50 | 0.000 | 0.017 | 0.037 | |
| ***School clubs measures*** | |  |  |  |  |  |  | |
| Number of academic clubs | | 0.003 | 0.009 | 0.35 | 0.723 | -0.015 | 0.021 | |
| Number of sports clubs | | 0.004 | 0.004 | 0.84 | 0.398 | -0.005 | 0.013 | |
| Number of arts clubs | | 0.055 | 0.011 | 5.15 | 0.000 | 0.034 | 0.076 | |
| Number of other clubs | | 0.063 | 0.008 | 7.77 | 0.000 | 0.047 | 0.079 | |
| ***School level variables*** | |  |  |  |  |  |  | |
| School dropout rate | | -0.005 | 0.002 | -2.15 | 0.031 | -0.009 | -0.000 | |
| Catholic school | | 0.536 | 0.140 | 3.83 | 0.000 | 0.262 | 0.811 | |
| Private school | | 0.271 | 0.159 | 1.71 | 0.088 | -0.040 | 0.583 | |
| Average distance to school | | 0.237 | 0.160 | 1.48 | 0.138 | -0.076 | 0.550 | |
| Standard deviation of distance between students in school | | 0.000 | 0.000 | 0.99 | 0.322 | -0.000 | 0.000 | |
| Average distance between students in school | | -0.188 | 0.123 | -1.53 | 0.126 | -0.429 | 0.053 | |
| ***School network measures*** | |  |  |  |  |  |  | |
| Density | | 0.065 | 0.313 | 0.21 | 0.835 | -0.548 | 0.678 | |
| Mutuality index | | 0.619 | 0.633 | 0.98 | 0.328 | -0.621 | 1.859 | |
| Size of school | | -0.000 | 0.000 | -3.34 | 0.001 | -0.000 | -0.000 | |
| ***Personal network measures*** | |  |  |  |  |  |  | |
| In-degree | | 0.003 | 0.002 | 1.91 | 0.057 | -0.000 | 0.006 | |
| Bonacich centrality | | 0.111 | 0.026 | 4.32 | 0.000 | 0.061 | 0.162 | |
| Personal network density | | -0.050 | 0.044 | -1.13 | 0.257 | -0.137 | 0.036 | |
| ***Block group level variables*** | |  |  |  |  |  |  | |
| Economic inequality | | -0.000 | 0.000 | -5.45 | 0.000 | -0.000 | -0.000 | |
| Concentrated disadvantage | | -0.192 | 0.068 | -2.81 | 0.005 | -0.326 | -0.058 | |
| Residential stability | | 0.037 | 0.007 | 5.43 | 0.000 | 0.024 | 0.050 | |
| Population density | | 0.017 | 0.002 | 8.51 | 0.000 | 0.013 | 0.021 | |
| Proportion Black | | 0.020 | 0.013 | 1.52 | 0.128 | -0.006 | 0.046 | |
| Proportion Latinx | | -0.016 | 0.016 | -1.02 | 0.310 | -0.047 | 0.015 | |
| Proportion Asian | | -0.055 | 0.013 | -4.24 | 0.000 | -0.080 | -0.029 | |
| Proportion Other race | | -0.009 | 0.014 | -0.62 | 0.533 | -0.037 | 0.019 | |
| Racial/ethnic heterogeneity | | 0.013 | 0.019 | 0.72 | 0.470 | -0.023 | 0.050 | |
| Percent foreign born | | -0.020 | 0.014 | -1.46 | 0.143 | -0.047 | 0.007 | |
| ***Individual level variables*** | |  |  |  |  |  |  | |
| Female | | 0.465 | 0.012 | 38.44 | 0.000 | 0.441 | 0.488 | |
| Grade | | 0.126 | 0.007 | 18.44 | 0.000 | 0.113 | 0.139 | |
| Black | | -0.166 | 0.021 | -7.93 | 0.000 | -0.208 | -0.125 | |
| Latinx | | -0.236 | 0.034 | -7.03 | 0.000 | -0.302 | -0.170 | |
| Asian | | -0.080 | 0.033 | -2.46 | 0.014 | -0.145 | -0.016 | |
| Native American/Other/Mixed | | -0.074 | 0.017 | -4.31 | 0.000 | -0.108 | -0.041 | |
| Native born | | 0.187 | 0.023 | 8.29 | 0.000 | 0.143 | 0.232 | |
| Years in this school | | -0.073 | 0.006 | -12.62 | 0.000 | -0.085 | -0.062 | |
| Intercept | | -0.697 | 0.227 | -3.07 | 0.002 | -1.142 | -0.253 | |
| ***Random effects*** | |  |  |  |  |  |  | |
| Variance Level 2 (Random Intercept) | | 0.079 | 0.011 |  |  | 0.060 | 0.104 | |
| ***Model fit statistics^a^*** | |  |  |  |  |  |  | |
| Log Likelihood | | -120105 |  |  |  |  |  | |
| Wald chi-square (*df*) | | 8313.18 (38) |  |  | 0.000 |  |  | |
| Number of observations | | 81,674 |  |  |  |  |  | |
| Number of groups (schools) | | 126 |  |  |  |  |  | |
| *Note*. Values estimated using a mixed effects negative binomial regression. Average distance to school and average distance between students in school measures rescaled (divided by 100,000). | | | | | | | | |
| ^a^ ICC estimate from a linear mixed model is 0.036 (standard error = 0.005). | | | | | | | | |
