# Supplementary material for: A multi-contextual examination of non-school friendships and their impact on adolescent deviance and alcohol use
Source: PLoS One. 2021 Feb 10;16(2):e0245837. doi: 10.1371/journal.pone.0245837 (PMC7875427; doi:10.1371/journal.pone.0245837)
Supplement: S2 Table — (DOCX) [file pone.0245837.s002.docx]

| **S2 Table. Results from MLM predicting alcohol use (no out of school ties)** | | | | |  |  |
| --- | --- | --- | --- | --- | --- | --- |
|  | Estimate | Standard Error | *z* | *p* | 95% *CI* | |
| Ties inside school | 0.015 | 0.002 | 6.17 | 0.000 | 0.010 | 0.019 |
| ***Parental measures*** |  |  |  |  |  |  |
| Parental monitoring | -1.515 | 0.063 | -24.11 | 0.000 | -1.638 | -1.392 |
| Parental support | -0.669 | 0.025 | -27.00 | 0.000 | -0.717 | -0.620 |
| ***School level variables*** |  |  |  |  |  |  |
| School dropout rate | 0.008 | 0.002 | 3.52 | 0.000 | 0.004 | 0.013 |
| ***Block group level variables*** |  |  |  |  |  |  |
| Concentrated disadvantage | 0.241 | 0.068 | 3.54 | 0.000 | 0.107 | 0.374 |
| ***Individual level variables*** |  |  |  |  |  |  |
| Female | -0.308 | 0.014 | -22.67 | 0.000 | -0.335 | -0.281 |
| Grade | 0.184 | 0.008 | 23.08 | 0.000 | 0.169 | 0.200 |
| Black | -0.361 | 0.024 | -14.90 | 0.000 | -0.409 | -0.314 |
| Latino | 0.117 | 0.038 | 3.07 | 0.002 | 0.042 | 0.191 |
| Asian | -0.755 | 0.041 | -18.56 | 0.000 | -0.835 | -0.676 |
| Native American/Other/Mixed | 0.130 | 0.020 | 6.60 | 0.000 | 0.091 | 0.168 |
| Native Born | 0.304 | 0.027 | 11.18 | 0.000 | 0.251 | 0.357 |
| School Attachment | -0.086 | 0.002 | -37.49 | 0.000 | -0.090 | -0.081 |
| Years in School | 0.052 | 0.007 | 7.63 | 0.000 | 0.039 | 0.066 |
| Cutpoint 1 | 1.051 | 0.083 | 12.70 | 0.000 | 0.889 | 1.213 |
| Cutpoint 2 | 2.232 | 0.083 | 26.86 | 0.000 | 2.070 | 2.395 |
| Cutpoint 3 | 2.810 | 0.083 | 33.71 | 0.000 | 2.646 | 2.973 |
| Cutpoint 4 | 3.562 | 0.084 | 42.54 | 0.000 | 3.398 | 3.727 |
| Cutpoint 5 | 4.695 | 0.085 | 55.31 | 0.000 | 4.529 | 4.861 |
| Cutpoint 6 | 5.446 | 0.087 | 62.84 | 0.000 | 5.276 | 5.616 |
| ***Random effects*** |  |  |  |  |  |  |
| Variance Level 2 (Random Intercept) | 0.121 | 0.019 |  |  | 0.090 | 0.164 |
| ***Model fit statistics^a^*** |  |  |  |  |  |  |
| Log Likelihood | -115546.23 |  |  |  |  |  |
| Wald chi-square (*df*) | 6395.37 (14) |  |  | 0.000 |  |  |
| Number of observations | 81,674 |  |  |  |  |  |
| Number of groups (schools) | 126 |  |  |  |  |  |
| *Note.* Values estimated using a mixed effects ordered logistic regression. | | | |  |  |  |
| ^a^ ICC estimate from a linear mixed model is 0.024 (standard error = 0.003). | | | |  |  |  |
