# Supplementary material for: A multi-contextual examination of non-school friendships and their impact on adolescent deviance and alcohol use
Source: PLoS One. 2021 Feb 10;16(2):e0245837. doi: 10.1371/journal.pone.0245837 (PMC7875427; doi:10.1371/journal.pone.0245837)
Supplement: S3 Table — (DOCX) [file pone.0245837.s003.docx]

| **S3 Table. Results from MLM predicting alcohol use with out of school ties** | | | | |  |  |
| --- | --- | --- | --- | --- | --- | --- |
|  | Estimate | Standard Error | *z* | *p* | 95% *CI* | |
| Ties inside school | 0.026 | 0.002 | 10.64 | 0.000 | 0.021 | 0.031 |
| Ties outside school | 0.066 | 0.003 | 19.35 | 0.000 | 0.059 | 0.072 |
| ***Parental measures*** |  |  |  |  |  |  |
| Parental monitoring | -1.471 | 0.063 | -23.39 | 0.000 | -1.594 | -1.348 |
| Parental support | -0.662 | 0.025 | -26.72 | 0.000 | -0.710 | -0.613 |
| ***School level variables*** |  |  |  |  |  |  |
| School dropout rate | 0.009 | 0.002 | 3.85 | 0.000 | 0.004 | 0.014 |
| ***Block group level variables*** |  |  |  |  |  |  |
| Concentrated disadvantage | 0.255 | 0.068 | 3.75 | 0.000 | 0.122 | 0.389 |
| ***Individual level variables*** |  |  |  |  |  |  |
| Female | -0.348 | 0.014 | -25.27 | 0.000 | -0.374 | -0.321 |
| Grade | 0.174 | 0.008 | 21.67 | 0.000 | 0.158 | 0.189 |
| Black | -0.352 | 0.024 | -14.49 | 0.000 | -0.399 | -0.304 |
| Latino | 0.140 | 0.038 | 3.67 | 0.000 | 0.065 | 0.214 |
| Asian | -0.749 | 0.041 | -18.37 | 0.000 | -0.829 | -0.669 |
| Native American/Other/Mixed | 0.137 | 0.020 | 6.95 | 0.000 | 0.098 | 0.175 |
| Native Born | 0.287 | 0.027 | 10.55 | 0.000 | 0.234 | 0.340 |
| School Attachment | -0.083 | 0.002 | -36.11 | 0.000 | -0.087 | -0.078 |
| Years in School | 0.060 | 0.007 | 8.74 | 0.000 | 0.047 | 0.074 |
| Cutpoint 1 | 1.120 | 0.083 | 13.48 | 0.000 | 0.957 | 1.283 |
| Cutpoint 2 | 2.307 | 0.084 | 27.62 | 0.000 | 2.143 | 2.470 |
| Cutpoint 3 | 2.886 | 0.084 | 34.45 | 0.000 | 2.722 | 3.050 |
| Cutpoint 4 | 3.641 | 0.084 | 43.26 | 0.000 | 3.476 | 3.806 |
| Cutpoint 5 | 4.775 | 0.085 | 55.97 | 0.000 | 4.607 | 4.942 |
| Cutpoint 6 | 5.526 | 0.087 | 63.47 | 0.000 | 5.355 | 5.696 |
| ***Random effects*** |  |  |  |  |  |  |
| Variance Level 2 (Random Intercept) | 0.125 | 0.019 |  |  | 0.093 | 0.168 |
| ***Model fit statistics^a^*** |  |  |  |  |  |  |
| Log Likelihood | -115360.08 |  |  |  |  |  |
| Wald chi-square (*df*) | 6740.08 (15) |  |  | 0.000 |  |  |
| Number of observations | 81,674 |  |  |  |  |  |
| Number of groups (schools) | 126 |  |  |  |  |  |
| *Note.* Values estimated using a mixed effects ordered logistic regression. | | | |  |  |  |
| ^a^ ICC estimate from a linear mixed model is 0.025 (standard error = 0.004). | | | |  |  |  |
