# Supplementary material for: A multi-contextual examination of non-school friendships and their impact on adolescent deviance and alcohol use
Source: PLoS One. 2021 Feb 10;16(2):e0245837. doi: 10.1371/journal.pone.0245837 (PMC7875427; doi:10.1371/journal.pone.0245837)
Supplement: S4 Table — (DOCX) [file pone.0245837.s004.docx]

| **S4 Table. Results from MLM predicting deviance (no out of school ties)** | | | | |  |  |
| --- | --- | --- | --- | --- | --- | --- |
|  | Estimate | Standard Error | *z* | *p* | 95% *CI* | |
| Ties inside school | -0.001 | 0.000 | -1.89 | 0.058 | -0.001 | 0.000 |
| ***Parental measures*** |  |  |  |  |  |  |
| Parental monitoring | -0.145 | 0.010 | -14.51 | 0.000 | -0.165 | -0.125 |
| Parental support | -0.184 | 0.004 | -46.41 | 0.000 | -0.192 | -0.176 |
| ***School level variables*** |  |  |  |  |  |  |
| School dropout rate | 0.001 | 0.000 | 4.50 | 0.000 | 0.001 | 0.002 |
| ***Block group level variables*** |  |  |  |  |  |  |
| Concentrated disadvantage | 0.067 | 0.011 | 6.14 | 0.000 | 0.045 | 0.088 |
| ***Individual level variables*** |  |  |  |  |  |  |
| Female | -0.075 | 0.002 | -34.59 | 0.000 | -0.079 | -0.071 |
| Grade | -0.006 | 0.001 | -4.67 | 0.000 | -0.008 | -0.003 |
| Black | -0.026 | 0.004 | -6.65 | 0.000 | -0.033 | -0.018 |
| Latino | 0.029 | 0.006 | 4.80 | 0.000 | 0.017 | 0.041 |
| Asian | -0.047 | 0.006 | -7.81 | 0.000 | -0.059 | -0.035 |
| Native American/Other/Mixed | 0.038 | 0.003 | 12.04 | 0.000 | 0.032 | 0.044 |
| Native Born | 0.026 | 0.004 | 6.29 | 0.000 | 0.018 | 0.035 |
| School Attachment | -0.021 | 0.000 | -57.47 | 0.000 | -0.021 | -0.020 |
| Years in School | 0.014 | 0.001 | 13.34 | 0.000 | 0.012 | 0.016 |
| Intercept | 0.224 | 0.013 | 17.72 | 0.000 | 0.199 | 0.249 |
| ***Random effects*** |  |  |  |  |  |  |
| Variance Level 1 (Residuals) | 0.091 | 0.000 |  |  | 0.090 | 0.092 |
| Variance Level 2 (Random Intercept) | 0.002 | 0.000 |  |  | 0.002 | 0.003 |
| ***Model fit statistics*** |  |  |  |  |  |  |
| Log Likelihood | -18146.03 |  |  |  |  |  |
| Wald chi-square (*df*) | 8680.39 (14) |  |  | 0.000 |  |  |
| Intraclass Correlation Coefficient (ICC) | 0.025 | 0.003 |  |  | 0.019 | 0.032 |
| Number of observations | 81,674 |  |  |  |  |  |
| Number of groups (schools) | 126 |  |  |  |  |  |
| *Note.* Values estimated using a mixed effects linear model. | | | |  |  |  |
