# Supplementary material for: A multi-contextual examination of non-school friendships and their impact on adolescent deviance and alcohol use
Source: PLoS One. 2021 Feb 10;16(2):e0245837. doi: 10.1371/journal.pone.0245837 (PMC7875427; doi:10.1371/journal.pone.0245837)
Supplement: S5 Table — (DOCX) [file pone.0245837.s005.docx]

| **S5 Table. Results from MLM predicting deviance with out of school ties** | | | | |  |  |
| --- | --- | --- | --- | --- | --- | --- |
|  | Estimate | Standard Error | *z* | *p* | 95% *CI* | |
| Ties inside school | 0.000 | 0.000 | 0.99 | 0.323 | -0.000 | 0.001 |
| Ties outside school | 0.007 | 0.001 | 12.12 | 0.000 | 0.006 | 0.008 |
| ***Parental measures*** |  |  |  |  |  |  |
| Parental monitoring | -0.140 | 0.010 | -14.04 | 0.000 | -0.160 | -0.121 |
| Parental support | -0.183 | 0.004 | -46.23 | 0.000 | -0.191 | -0.175 |
| ***School level variables*** |  |  |  |  |  |  |
| School dropout rate | 0.002 | 0.000 | 4.71 | 0.000 | 0.001 | 0.002 |
| ***Block group level variables*** |  |  |  |  |  |  |
| Concentrated disadvantage | 0.068 | 0.011 | 6.29 | 0.000 | 0.047 | 0.090 |
| ***Individual level variables*** |  |  |  |  |  |  |
| Female | -0.079 | 0.002 | -36.03 | 0.000 | -0.083 | -0.074 |
| Grade | -0.007 | 0.001 | -5.52 | 0.000 | -0.009 | -0.004 |
| Black | -0.024 | 0.004 | -6.36 | 0.000 | -0.032 | -0.017 |
| Latino | 0.031 | 0.006 | 5.15 | 0.000 | 0.019 | 0.043 |
| Asian | -0.046 | 0.006 | -7.67 | 0.000 | -0.058 | -0.035 |
| Native American/Other/Mixed | 0.039 | 0.003 | 12.26 | 0.000 | 0.032 | 0.045 |
| Native Born | 0.025 | 0.004 | 5.90 | 0.000 | 0.016 | 0.033 |
| School Attachment | -0.020 | 0.000 | -56.55 | 0.000 | -0.021 | -0.020 |
| Years in School | 0.015 | 0.001 | 14.00 | 0.000 | 0.013 | 0.017 |
| Intercept | 0.217 | 0.013 | 17.14 | 0.000 | 0.192 | 0.242 |
| ***Random effects*** |  |  |  |  |  |  |
| Variance Level 1 (Residual) | 0.091 | 0.000 |  |  | 0.090 | 0.092 |
| Variance Level 2 (Random Intercept) | 0.002 | 0.000 |  |  | 0.002 | 0.003 |
| ***Model fit statistics*** |  |  |  |  |  |  |
| Log Likelihood | -18072.70 |  |  |  |  |  |
| Wald chi-square (d*f*) | 8841.49 (15) |  |  | 0.000 |  |  |
| Intraclass Correlation Coefficient (ICC) | 0.025 | 0.003 |  |  | 0.019 | 0.033 |
| Number of observations | 81,674 |  |  |  |  |  |
| Number of groups (schools) | 126 |  |  |  |  |  |
| *Note.* Values estimated using a mixed effects linear model. | | | |  |  |  |
