# Supplementary material for: A multi-contextual examination of non-school friendships and their impact on adolescent deviance and alcohol use
Source: PLoS One. 2021 Feb 10;16(2):e0245837. doi: 10.1371/journal.pone.0245837 (PMC7875427; doi:10.1371/journal.pone.0245837)
Supplement: S6 Table — (DOCX) [file pone.0245837.s006.docx]

| **S6 Table. Results from MLM predicting out of school friendships with public school type x White race** | | | | | | | |
| --- | --- | --- | --- | --- | --- | --- | --- |
|  |  | Estimate | Standard Error | *z* | *p* | 95% *CI* | |
| Ties inside school | | -0.200 | 0.006 | -35.33 | 0.000 | -0.211 | -0.189 |
| ***Parental measures*** | |  |  |  |  |  |  |
| Parental monitoring | | -0.469 | 0.053 | -8.77 | 0.000 | -0.574 | -0.364 |
| Parental support | | -0.144 | 0.021 | -6.89 | 0.000 | -0.186 | -0.103 |
| Education (mother) | | 0.026 | 0.005 | 5.30 | 0.000 | 0.016 | 0.035 |
| ***School clubs measures*** | |  |  |  |  |  |  |
| Number of academic clubs | | 0.004 | 0.009 | 0.48 | 0.632 | -0.014 | 0.023 |
| Number of sports clubs | | 0.003 | 0.004 | 0.75 | 0.452 | -0.005 | 0.012 |
| Number of arts clubs | | 0.055 | 0.011 | 5.17 | 0.000 | 0.034 | 0.076 |
| Number of other clubs | | 0.065 | 0.008 | 7.92 | 0.000 | 0.049 | 0.081 |
| ***School level variables*** | |  |  |  |  |  |  |
| School dropout rate | | -0.005 | 0.002 | -2.23 | 0.026 | -0.009 | -0.001 |
| Public school | | -0.514 | 0.118 | -4.37 | 0.000 | -0.744 | -0.283 |
| Average distance to school | | 0.252 | 0.160 | 1.58 | 0.114 | -0.061 | 0.565 |
| Standard deviation of distance between students in school | | 0.000 | 0.000 | 1.14 | 0.253 | -0.000 | 0.000 |
| Average distance between students in school | | -0.202 | 0.123 | -1.65 | 0.099 | -0.442 | 0.038 |
| ***School network measures*** | |  |  |  |  |  |  |
| Density | | 0.061 | 0.313 | 0.20 | 0.844 | -0.551 | 0.674 |
| Mutuality index | | 0.623 | 0.632 | 0.99 | 0.324 | -0.616 | 1.862 |
| Size of school | | -0.000 | 0.000 | -3.19 | 0.001 | -0.000 | -0.000 |
| ***Personal network measures*** | |  |  |  |  |  |  |
| In-degree | | 0.003 | 0.002 | 1.90 | 0.057 | -0.000 | 0.006 |
| Bonacich centrality | | 0.113 | 0.026 | 4.40 | 0.000 | 0.063 | 0.164 |
| Personal network density | | -0.053 | 0.044 | -1.21 | 0.227 | -0.140 | 0.033 |
| ***Block group level variables*** | |  |  |  |  |  |  |
| Economic inequality | | -0.000 | 0.000 | -5.35 | 0.000 | -0.000 | -0.000 |
| Concentrated disadvantage | | -0.210 | 0.068 | -3.10 | 0.002 | -0.343 | -0.077 |
| Residential stability | | 0.035 | 0.007 | 5.19 | 0.000 | 0.022 | 0.048 |
| Population density | | 0.018 | 0.002 | 8.90 | 0.000 | 0.014 | 0.021 |
| Proportion Black | | 0.015 | 0.013 | 1.18 | 0.239 | -0.010 | 0.041 |
| Proportion Latinx | | -0.015 | 0.016 | -0.98 | 0.325 | -0.046 | 0.015 |
| Proportion Asian | | -0.050 | 0.013 | -3.90 | 0.000 | -0.075 | -0.025 |
| Proportion Other race | | -0.002 | 0.014 | -0.13 | 0.899 | -0.030 | 0.026 |
| Racial/ethnic heterogeneity | | 0.016 | 0.019 | 0.85 | 0.396 | -0.021 | 0.052 |
| Percent foreign born | | -0.020 | 0.014 | -1.44 | 0.149 | -0.046 | 0.007 |
| ***Individual level variables*** | |  |  |  |  |  |  |
| Female | | 0.462 | 0.012 | 38.29 | 0.000 | 0.438 | 0.486 |
| Grade | | 0.124 | 0.007 | 18.19 | 0.000 | 0.111 | 0.138 |
| White race | | 0.002 | 0.063 | 0.03 | 0.975 | -0.121 | 0.125 |
| Native born | | 0.171 | 0.022 | 7.89 | 0.000 | 0.129 | 0.214 |
| Years in this school | | -0.073 | 0.006 | -12.59 | 0.000 | -0.085 | -0.062 |
| ***Interaction*** | |  |  |  |  |  |  |
| Public school x White race | | 0.152 | 0.064 | 2.38 | 0.017 | 0.027 | 0.278 |
| Intercept | | -0.289 | 0.268 | -1.08 | 0.280 | -0.814 | 0.236 |
| ***Random effects*** | |  |  |  |  |  |  |
| Variance Level 2 (Random Intercept) | | 0.079 | 0.011 |  |  | 0.060 | 0.104 |
| ***Model fit statistics^a^*** | |  |  |  |  |  |  |
| Log Likelihood | | -120101.4 |  |  |  |  |  |
| Wald chi-square (*df*) | | 8321.96 (35) |  |  | 0.000 |  |  |
| Number of observations | | 81,674 |  |  |  |  |  |
| Number of groups (schools) | | 126 |  |  |  |  |  |
| *Note*. Values estimated using a mixed effects negative binomial regression. Average distance to school and average distance between students in school measures rescaled (divided by 100,000). | | | | | | | |
| ^a^ ICC estimate from a linear mixed model is 0.038 (standard error = 0.005). | | | | | | | |
