# Supplementary material for: A multi-contextual examination of non-school friendships and their impact on adolescent deviance and alcohol use
Source: PLoS One. 2021 Feb 10;16(2):e0245837. doi: 10.1371/journal.pone.0245837 (PMC7875427; doi:10.1371/journal.pone.0245837)
Supplement: S7 Table — (DOCX) [file pone.0245837.s007.docx]

| **S7 Table. Results from MLM predicting out of school friendships with parental monitoring x neighborhood inequality** | | | | | | | | |
| --- | --- | --- | --- | --- | --- | --- | --- | --- |
|  |  | Estimate | Standard Error | | *z* | *p* | 95% *CI* | |
| Ties inside school | | -0.200 | 0.006 | | -35.21 | 0.000 | -0.211 | -0.189 |
| ***Parental measures*** | |  |  | |  |  |  |  |
| Parental monitoring | | -0.465 | 0.054 | | -8.69 | 0.000 | -0.570 | -0.360 |
| Parental support | | -0.143 | 0.021 | | -6.81 | 0.000 | -0.184 | -0.102 |
| Education (mother) | | 0.027 | 0.005 | | 5.50 | 0.000 | 0.017 | 0.037 |
| ***School clubs measures*** | |  |  | |  |  |  |  |
| Number of academic clubs | | 0.003 | 0.009 | | 0.38 | 0.707 | -0.015 | 0.022 |
| Number of sports clubs | | 0.004 | 0.004 | | 0.84 | 0.402 | -0.005 | 0.013 |
| Number of arts clubs | | 0.055 | 0.011 | | 5.13 | 0.000 | 0.034 | 0.076 |
| Number of other clubs | | 0.063 | 0.008 | | 7.77 | 0.000 | 0.047 | 0.079 |
| ***School level variables*** | |  |  | |  |  |  |  |
| School dropout rate | | -0.005 | 0.002 | | -2.17 | 0.030 | -0.009 | -0.000 |
| Catholic school | | 0.537 | 0.140 | | 3.83 | 0.000 | 0.262 | 0.811 |
| Private school | | 0.271 | 0.159 | | 1.71 | 0.088 | -0.040 | 0.583 |
| Average distance to school | | 0.237 | 0.160 | | 1.48 | 0.138 | -0.076 | 0.551 |
| Standard deviation of distance between students in school | | 0.000 | 0.000 | | 0.99 | 0.320 | -0.000 | 0.000 |
| Average distance between students in school | | -0.188 | 0.123 | | -1.53 | 0.126 | -0.429 | 0.053 |
| ***School network measures*** | |  |  | |  |  |  |  |
| Density | | 0.067 | 0.313 | | 0.21 | 0.830 | -0.546 | 0.680 |
| Mutuality index | | 0.601 | 0.633 | | 0.95 | 0.342 | -0.639 | 1.842 |
| Size of school | | -0.000 | 0.000 | | -3.33 | 0.001 | -0.000 | -0.000 |
| ***Personal network measures*** | |  |  | |  |  |  |  |
| In-degree | | 0.003 | 0.002 | | 1.92 | 0.055 | -0.000 | 0.006 |
| Bonacich centrality | | 0.112 | 0.026 | | 4.33 | 0.000 | 0.061 | 0.162 |
| Personal network density | | -0.050 | 0.044 | | -1.14 | 0.254 | -0.137 | 0.036 |
| ***Block group level variables*** | |  |  | |  |  |  |  |
| Economic inequality | | -0.000 | 0.000 | | -5.46 | 0.000 | -0.000 | -0.000 |
| Concentrated disadvantage | | -0.192 | 0.068 | | -2.82 | 0.005 | -0.326 | -0.059 |
| Residential stability | | 0.037 | 0.007 | | 5.44 | 0.000 | 0.024 | 0.050 |
| Population density | | 0.017 | 0.002 | | 8.53 | 0.000 | 0.013 | 0.021 |
| Proportion Black | | 0.020 | 0.013 | | 1.51 | 0.131 | -0.006 | 0.046 |
| Proportion Latinx | | -0.016 | 0.016 | | -1.01 | 0.314 | -0.046 | 0.015 |
| Proportion Asian | | -0.054 | 0.013 | | -4.23 | 0.000 | -0.080 | -0.029 |
| Proportion Other race | | -0.009 | 0.014 | | -0.63 | 0.530 | -0.037 | 0.019 |
| Racial/ethnic heterogeneity | | 0.014 | 0.019 | | 0.73 | 0.463 | -0.023 | 0.050 |
| Percent foreign born | | -0.020 | 0.014 | | -1.45 | 0.146 | -0.047 | 0.007 |
| ***Individual level variables*** | |  |  | |  |  |  |  |
| Female | | 0.465 | 0.012 | | 38.45 | 0.000 | 0.441 | 0.488 |
| Grade | | 0.126 | 0.007 | | 18.46 | 0.000 | 0.113 | 0.139 |
| Black | | -0.166 | 0.021 | | -7.93 | 0.000 | -0.207 | -0.125 |
| Latinx | | -0.236 | 0.034 | | -7.01 | 0.000 | -0.302 | -0.170 |
| Asian | | -0.081 | 0.033 | | -2.46 | 0.014 | -0.145 | -0.016 |
| Native American/Other/Mixed | | -0.075 | 0.017 | | -4.32 | 0.000 | -0.108 | -0.041 |
| Native born | | 0.188 | 0.023 | | 8.32 | 0.000 | 0.144 | 0.232 |
| Years in this school | | -0.073 | 0.006 | | -12.61 | 0.000 | -0.085 | -0.062 |
| ***Interaction*** | |  |  | |  |  |  |  |
| Parental monitoring x Economic inequality | | 0.000 | 0.000 | | 2.38 | 0.017 | 0.000 | 0.000 |
| Intercept | | -0.692 | 0.227 | | -3.05 | 0.002 | -1.137 | -0.248 |
| ***Random effects*** | |  |  | |  |  |  |  |
| Variance Level 2 (Random Intercept) | | 0.079 | 0.011 | |  |  | 0.060 | 0.104 |
| ***Model fit statistics^a^*** | |  |  | |  |  |  |  |
| Log Likelihood | | -120102.16 |  | |  |  |  |  |
| Wald chi-square (*df*) | | 8317.53 (39) |  | |  | 0.000 |  |  |
| Number of observations | | 81,674 |  | |  |  |  |  |
| Number of groups (schools) | | 126 |  | |  |  |  |  |
| *Note*. Values estimated using a mixed effects negative binomial regression. Average distance to school and average distance between students in school measures rescaled (divided by 100,000). | | | | | | | | |
| ^a^ ICC estimate from a linear mixed model is 0.036 (standard error = 0.005). | | | |  |  |  |  |  |
