# Supplementary material for: A multi-contextual examination of non-school friendships and their impact on adolescent deviance and alcohol use
Source: PLoS One. 2021 Feb 10;16(2):e0245837. doi: 10.1371/journal.pone.0245837 (PMC7875427; doi:10.1371/journal.pone.0245837)
Supplement: S8 Table — (DOCX) [file pone.0245837.s008.docx]

| **S8 Table. Results from MLM predicting out of school friendships with parental monitoring x neighborhood disadvantage** | | | | | | | |
| --- | --- | --- | --- | --- | --- | --- | --- |
|  |  | Estimate | Standard Error | *z* | *p* | 95% *CI* | |
| Ties inside school | | -0.200 | 0.006 | -35.20 | 0.000 | -0.211 | -0.188 |
| ***Parental measures*** | |  |  |  |  |  |  |
| Parental monitoring | | -0.467 | 0.054 | -8.72 | 0.000 | -0.572 | -0.362 |
| Parental support | | -0.142 | 0.021 | -6.79 | 0.000 | -0.184 | -0.101 |
| Education (mother) | | 0.027 | 0.005 | 5.51 | 0.000 | 0.017 | 0.037 |
| ***School clubs measures*** | |  |  |  |  |  |  |
| Number of academic clubs | | 0.004 | 0.009 | 0.40 | 0.691 | -0.015 | 0.022 |
| Number of sports clubs | | 0.004 | 0.004 | 0.85 | 0.397 | -0.005 | 0.013 |
| Number of arts clubs | | 0.055 | 0.011 | 5.13 | 0.000 | 0.034 | 0.076 |
| Number of other clubs | | 0.063 | 0.008 | 7.74 | 0.000 | 0.047 | 0.079 |
| ***School level variables*** | |  |  |  |  |  |  |
| School dropout rate | | -0.005 | 0.002 | -2.18 | 0.030 | -0.009 | -0.000 |
| Catholic school | | 0.536 | 0.140 | 3.82 | 0.000 | 0.261 | 0.811 |
| Private school | | 0.270 | 0.159 | 1.70 | 0.090 | -0.042 | 0.582 |
| Average distance to school | | 0.239 | 0.160 | 1.49 | 0.135 | -0.075 | 0.553 |
| Standard deviation of distance between students in school | | 0.000 | 0.000 | 1.00 | 0.319 | -0.000 | 0.000 |
| Average distance between students in school | | -0.189 | 0.123 | -1.54 | 0.124 | -0.431 | 0.052 |
| ***School network measures*** | |  |  |  |  |  |  |
| Density | | 0.062 | 0.313 | 0.20 | 0.842 | -0.551 | 0.676 |
| Mutuality index | | 0.600 | 0.633 | 0.95 | 0.343 | -0.641 | 1.842 |
| Size of school | | -0.000 | 0.000 | -3.33 | 0.001 | -0.000 | -0.000 |
| ***Personal network measures*** | |  |  |  |  |  |  |
| In-degree | | 0.003 | 0.002 | 1.92 | 0.055 | -0.000 | 0.006 |
| Bonacich centrality | | 0.111 | 0.026 | 4.31 | 0.000 | 0.061 | 0.162 |
| Personal network density | | -0.050 | 0.044 | -1.14 | 0.253 | -0.137 | 0.036 |
| ***Block group level variables*** | |  |  |  |  |  |  |
| Economic inequality | | -0.000 | 0.000 | -5.44 | 0.000 | -0.000 | -0.000 |
| Concentrated disadvantage | | -0.191 | 0.068 | -2.79 | 0.005 | -0.324 | -0.057 |
| Residential stability | | 0.037 | 0.007 | 5.44 | 0.000 | 0.024 | 0.050 |
| Population density | | 0.017 | 0.002 | 8.51 | 0.000 | 0.013 | 0.021 |
| Proportion Black | | 0.020 | 0.013 | 1.52 | 0.129 | -0.006 | 0.046 |
| Proportion Latinx | | -0.016 | 0.016 | -1.01 | 0.311 | -0.047 | 0.015 |
| Proportion Asian | | -0.055 | 0.013 | -4.25 | 0.000 | -0.080 | -0.029 |
| Proportion Other race | | -0.009 | 0.014 | -0.62 | 0.538 | -0.037 | 0.019 |
| Racial/ethnic heterogeneity | | 0.013 | 0.019 | 0.71 | 0.477 | -0.023 | 0.050 |
| Percent foreign born | | -0.020 | 0.014 | -1.45 | 0.148 | -0.047 | 0.007 |
| ***Individual level variables*** | |  |  |  |  |  |  |
| Female | | 0.465 | 0.012 | 38.43 | 0.000 | 0.441 | 0.488 |
| Grade | | 0.126 | 0.007 | 18.49 | 0.000 | 0.113 | 0.140 |
| Black | | -0.166 | 0.021 | -7.92 | 0.000 | -0.207 | -0.125 |
| Latinx | | -0.236 | 0.034 | -7.02 | 0.000 | -0.302 | -0.170 |
| Asian | | -0.080 | 0.033 | -2.45 | 0.014 | -0.144 | -0.016 |
| Native American/Other/Mixed | | -0.075 | 0.017 | -4.32 | 0.000 | -0.108 | -0.041 |
| Native born | | 0.187 | 0.023 | 8.29 | 0.000 | 0.143 | 0.232 |
| Years in this school | | -0.073 | 0.006 | -12.63 | 0.000 | -0.085 | -0.062 |
| ***Interaction*** | |  |  |  |  |  |  |
| Parental monitoring x Concentrated disadvantage | | -1.232 | 0.414 | -2.98 | 0.003 | -2.043 | -0.421 |
| Intercept | | -0.690 | 0.227 | -3.04 | 0.002 | -1.135 | -0.245 |
| ***Random effects*** | |  |  |  |  |  |  |
| Variance Level 2 (Random Intercept) | | 0.079 | 0.011 |  |  | 0.060 | 0.104 |
| ***Model fit statistics^a^*** | |  |  |  |  |  |  |
| Log Likelihood | | -120100.57 |  |  |  |  |  |
| Wald chi-square (*df*) | | 8321.49 (39) |  |  | 0.000 |  |  |
| Number of observations | | 81,674 |  |  |  |  |  |
| Number of groups (schools) | | 126 |  |  |  |  |  |
| *Note*. Values estimated using a mixed effects negative binomial regression. Average distance to school and average distance between students in school measures rescaled (divided by 100,000). | | | | | | | |
| ^a^ ICC estimate from a linear mixed model is 0.036 (standard error = 0.005). | | | | | | | |
