# Supplementary figures and images for: A multi-contextual examination of non-school friendships and their impact on adolescent deviance and alcohol use
Source: PLoS One. 2021 Feb 10;16(2):e0245837. doi: 10.1371/journal.pone.0245837 (PMC7875427; doi:10.1371/journal.pone.0245837)

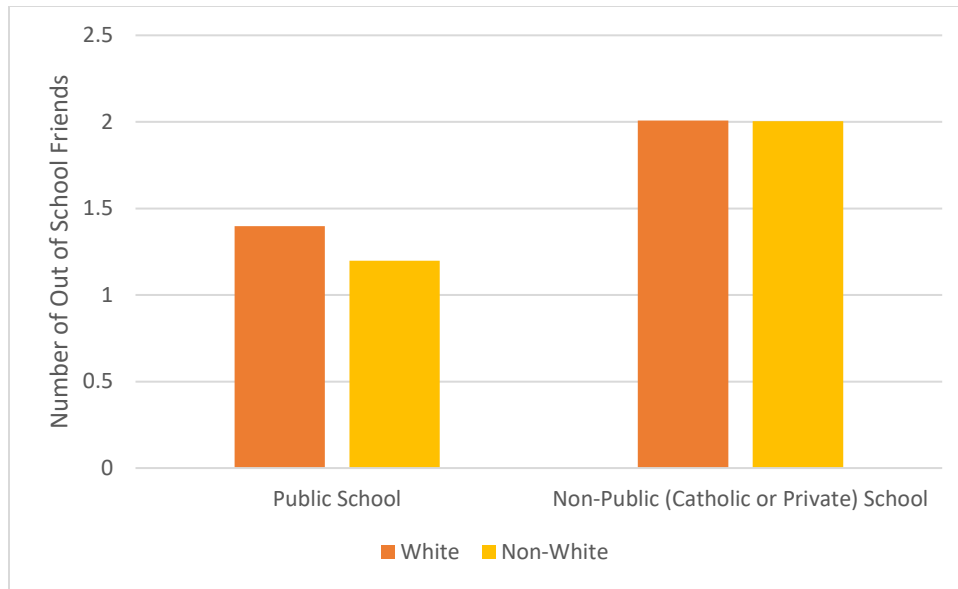

**Figure S1. Predicting out of school ties: Public school type x White race interaction**

Supplement: S1 Fig — (PDF) [file pone.0245837.s009.pdf]
